# Systemic lipid and glucose modulation differentially affects cognitive function and neuroinflammation in a mouse model of Alzheimer’s disease

**DOI:** 10.3389/fnins.2025.1636624

**Published:** 2025-08-29

**Authors:** Demos Kynigopoulos, Eleni Fella, Lucy Shahabian, Christiana C. Christodoulou, Revekka Papacharalampous, Konstantinos Diskos, Lida Evmorfia Vagiaki, Kyriaki Sidiropoulou, Menelaos Pipis, Kleopas A. Kleopa, Elena Panayiotou

**Affiliations:** ^1^Neuropathology Department, The Cyprus Institute of Neurology and Genetics, Nicosia, Cyprus; ^2^Neuroepidemiology Department, The Cyprus Institute of Neurology and Genetics, Nicosia, Cyprus; ^3^Department of Biology, University of Crete, Heraklion, Greece; ^4^Institute of Molecular Biology and Biotechnology, Foundation for Research and Technology-Hellas (IMBB-FORTH), Heraklion, Greece; ^5^Department of Neuroscience, The Cyprus Institute of Neurology and Genetics, Nicosia, Cyprus

**Keywords:** Alzheimer’s disease, lipid metabolism, glucose metabolism, synaptic plasticity, 5xFAD mouse model, Alirocumab, Gliclazide, neuroinflammation

## Abstract

**Introduction:**

Alzheimer’s disease (AD) is a progressive neurodegenerative disorder characterized by synaptic dysfunction and cognitive decline. Increasing evidence implicates systemic metabolic dysregulation in AD pathogenesis, yet it remains unclear whether modulation of peripheral lipid and glucose metabolism can alter disease progression.

**Methods:**

We investigated the effects of two FDA-approved metabolic agents—Alirocumab, a PCSK9 inhibitor that lowers LDL cholesterol, and Gliclazide, a sulfonylurea that enhances insulin secretion—in male 5xFAD mice, a transgenic model of AD. Animals received chronic treatment for five months. Behavioral testing, hippocampal electrophysiology, ELISA, lipidomics, and adipokine profiling were performed to assess cognitive, synaptic, and molecular outcomes.

**Results:**

Alirocumab significantly improved spatial working memory, restored hippocampal long-term potentiation, and normalized synaptophysin expression. Gliclazide reduced neuroinflammation and partially preserved glial and neuronal markers. Both treatments decreased amyloid burden and modulated adipokine levels, with Alirocumab elevating leptin and omentin in brain and serum. Lipidomic profiling of visceral adipose tissue revealed distinct lipid remodeling and highlighted candidate pathways linking systemic metabolism to central nervous system outcomes.

**Discussion:**

These findings demonstrate that systemic modulation of lipid and glucose metabolism can influence neurodegenerative and synaptic processes in AD. The results support metabolic interventions as a potential strategy to modify AD progression through peripheral–central metabolic crosstalk.

## Introduction

Alzheimer’s disease (AD) is a progressive neurodegenerative disorder closely linked to aging and characterized by the accumulation of extracellular *β*-amyloid plaques and intracellular neurofibrillary tangles composed of hyperphosphorylated tau protein (Alzheimer, 1911). These hallmark features are associated with synaptic dysfunction, cognitive decline, and persistent neuroinflammation (Knopman et al., 2021). The pathogenesis of AD involves a multifaceted interaction of genetic, environmental, and metabolic factors (Liu et al., 2024), highlighting the need for comprehensive therapeutic approaches that address the disease’s complexity (Gaugler et al., 2024).

Lipids are essential for maintaining brain health, contributing to the stability of the blood–brain barrier (BBB), regulation of amyloid precursor protein (APP) processing, synaptic signaling, and overall energy balance in the central nervous system (Yin, 2023). Disruptions in lipid metabolism are increasingly recognized in AD. In particular, disturbances in cholesterol synthesis, transport, and breakdown have been linked to increased amyloid production and impaired synaptic function (Livingston et al., 2024; Feringa and van der Kant, 2021). Cholesterol-derived metabolites such as oxysterols are being explored for their potential role in disease progression (Wingo et al., 2019; Wingo et al., 2022; Varma et al., 2021; Shippy and Ulland, 2023). Supporting this, animal studies have shown that statins, including atorvastatin, can reduce brain inflammation and improve cognitive performance in AD models (Zhao et al., 2016).

Metabolic dysfunction, especially in the context of Type 2 Diabetes (T2D), has also been strongly associated with AD. The Rotterdam Study reported a two-fold increase in AD risk among individuals with T2D (Ahmadizar et al., 2021), while subsequent studies have shown a 65% higher risk compared to non-diabetic populations (Ott et al., 1999). Experimental models reinforce this link: mice lacking insulin and insulin-like growth factor 1 (IGF-1) receptors exhibit both glucose intolerance and cognitive impairment (Soto et al., 2019), and AD mice crossbred with diabetic strains display lower insulin levels and more rapid cognitive decline (King et al., 2020). These findings underscore the importance of systemic metabolic health in shaping neurodegenerative outcomes.

Genome-wide association studies (GWAS) have further identified metabolic factors—including altered lipid handling, insulin resistance, and elevated blood glucose—as key contributors to cognitive aging and disease risk (Chia et al., 2018). Nutritional interventions aimed at lowering carbohydrate intake have been proposed to support brain health and maintain the expression of genes involved in healthy aging (Rubio-Tomás et al., 2022). Emerging evidence also suggests a two-way relationship between AD and T2D, where each condition may worsen the other (Pruzin et al., 2018; Fiore et al., 2019). While impaired insulin signaling plays a major role, lipid metabolism—particularly involving fatty acid handling and apolipoprotein E (ApoE) function—also appears to contribute significantly to both metabolic and neurodegenerative processes (Fortea et al., 2024). In the absence of effective disease-modifying treatments for AD, repurposing existing metabolic drugs offers a practical and promising therapeutic direction.

In this study, we examined the effects of two FDA-approved metabolic drugs, Alirocumab, a PCSK9 inhibitor, and Gliclazide, a sulfonylurea, using the 5xFAD mouse model, which develops early and aggressive amyloid pathology. Our aim was to evaluate whether targeting peripheral lipid and glucose metabolism could impact central nervous system outcomes, including synaptic function, cognitive performance, and hallmark AD pathology. We also sought to identify potential biomarkers and mechanisms linking systemic metabolism with brain health. Alirocumab lowers low-density lipoprotein cholesterol (LDL-C) by inhibiting PCSK9, thereby enhancing hepatic LDL receptor availability and cholesterol clearance (Manniello and Pisano, 2016; O’Connell and Lohoff, 2020; Mazura et al., 2022). Gliclazide improves blood glucose control by stimulating insulin secretion from pancreatic *β*-cells and is widely used in the treatment of T2D (Al-Omary, 2017; Ibrahim and Hassan, 2018). By leveraging these two mechanistically distinct agents, we explored whether metabolic interventions could influence AD progression and offer new opportunities for therapeutic development.

## Materials and methods

### Animals and handling

The 5xFAD mouse model is a transgenic line that co-expresses human APP and PSEN1 genes carrying familial Alzheimer’s disease (FAD) mutations. Specifically, it incorporates three APP mutations, the Swedish (K670N/M671L), Florida (I716V), and London (V717I) variants and two PSEN1 mutations (M146L and L286V), all under the transcriptional control of the neuron-specific murine Thy-1 promoter (Tg6799 line; Oakley et al., 2006). The Swedish mutation increases total amyloid-*β* (Aβ) levels, while the Florida and London mutations enhance the generation of the Aβ₄₂ isoform, which is more prone to aggregation. All 5xFAD mice were maintained on a hybrid B6/SJL background and were heterozygous for the transgenes. To generate experimental cohorts, male heterozygous 5xFAD mice—originally obtained as littermates—were bred with wild-type B6/SJL females. Non-transgenic wild-type (WT) littermates served as controls. This model exhibits early and aggressive amyloid pathology, with initial deposition in the cortex and hippocampus, followed by progressive involvement of additional brain regions.

In the 5xFAD mouse model, intraneuronal Aβ42 aggregates appear by 1.5 months, followed by amyloid plaque deposition in the subiculum and cortical layer V by 2 months, with females showing greater pathology (Maarouf et al., 2013; Bhattacharya et al., 2014; Sadleir et al., 2015). Gliosis begins concurrently, and by 4 months, synaptic loss is evident, marked by decreased synaptophysin. Cognitive deficits in spatial and remote memory emerge at 4–5 months. Between 4 and 6 months, impairments in CA1 LTP and basal synaptic transmission are observed (Kimura and Ohno, 2009; Crouzin et al., 2013). Neuronal loss becomes apparent by 6 months in cortical and subicular regions, and reductions in syntaxin and PSD-95 occur by 9 months (Oakley et al., 2006). Notably, tau pathology is absent in this model (Oakley et al., 2006).

For the experiments, only male mice were used. Animals were kept in separate cages based on the treatment group they belonged to. Mice were housed in groups of up to five animals per cage.

Genotyping was performed by extracting DNA from tail biopsies, followed by PCR using primers specific for human APP (F: 5′-AGG ACT GAC CAC TCG ACC AG-3′, R: 5’-CGG GGG TCT AGT TCT GCA T-3′) and PSEN1 (F: 5′-AAT AGA GAA CGG CAG GAG CA-3′, R: 5’-GCC ATG AGG GCA CTA ATC AT-3′). PCR products were resolved on a 1.5% agarose gel and visualized under UV light to distinguish wild-type and 5xFAD genotypes.

**Ethics statement**: The animals were kept in a regular 12 h light/12 h dark cycle during the experimental phase and they were allowed to consume food and water ad libitum. All animals were kept and handled under specific pathogen-free (SPF) conditions following the 86/609/EEC Directive which certifies the appropriate treatment of animals.

### Animal groups and treatment

The total number of animals were 67. The animals were sorted into 6 groups (WT untreated: *N* = 9, WT Gliclazide: *N* = 12, WT Alirocumab: *N* = 12, 5xFAD untreated: *N* = 10, 5xFAD Gliclazide: *N* = 12, 5xFAD Alirocumab: *N* = 12). No animals were excluded from the experiments.

Drug administration was started at the age of 3-months. The Gliclazide was administered by oral gavage every other day (Mon, Wed, Fri) at a concentration of 20 mg/Kg (Vallejo et al., 2000; Sarkar et al., 2011). The Alirocumab was administered by subcutaneous injection every 10 days at a concentration of 10 mg/Kg (Greig and Deeks, 2016; Ramanathan et al., 2016). The treatment had a duration of 5 months. At 8 months of age, the mice stopped receiving any treatment (Supplementary Table 1). All experimental procedures were performed on all the male 5xFAD and wild-type littermates at 8 months of age.

### Behavioral testing

At the end of the treatment period (8 months of age), mice underwent the Y-maze spontaneous alternation task to evaluate cognitive function, specifically working memory and spatial learning. Each animal was placed at the center of the Y-shaped maze and allowed to freely explore all three arms for a duration of 6 mins. This task assesses the animals’ ability to recall and alternate between previously visited arms, reflecting short-term spatial memory performance. A reduced alternation rate is indicative of cognitive impairment and has been associated with various neurological and psychiatric conditions (Kraeuter et al., 2019). Spontaneous alternation was calculated as the percentage of successful triads, defined as consecutive entries into all three arms without repetition, divided by the total possible triads (total arm entries minus two), multiplied by 100.

### Electrophysiology

The experimental procedures described below were followed to investigate and measure the effects of various stimuli on synaptic activity in the mouse brain slices obtained.

Mice were euthanized under halothane anesthesia, and brains were rapidly removed and immersed in ice-cold, oxygenated artificial cerebrospinal fluid (aCSF; 95% O₂/5% CO₂) containing 124 mM NaCl, 3 mM KCl, 26 mM NaHCO₃, 1 mM MgSO₄, 1.25 mM NaH₂PO₄, and 10 mM glucose (pH 7.4, 310 mOsm/L). Coronal brain slices (400 μm) containing the hippocampus were prepared from 8-month-old male mice using a vibratome (Leica VT1000S) and transferred to a recording chamber containing oxygenated aCSF (with 2 mM CaCl₂ and 1 mM MgCl₂) at 32–34 °C for at least 1 h before recordings (Velli et al., 2021).

Field excitatory postsynaptic potentials (fEPSPs) were recorded in the CA1 stratum radiatum using glass microelectrodes filled with 3 M NaCl (Harvard Apparatus), and Schaffer collaterals were stimulated with platinum/iridium microelectrodes placed ~300–400 μm apart. Signals were amplified (EXT-02F), digitized (ITC-18 board), and recorded using IgorPro (Wavemetrics) with custom acquisition scripts.

Stimulation consisted of single 100 μs square pulses (0.1–0.4 mA) delivered via a stimulus isolation unit (WPI). fEPSP amplitude was measured from the point of deflection to 3–5 ms post-stimulus. Input–output curves were generated by averaging two traces per intensity, and baseline stimulation was set to evoke ~1 mV responses.

Long-term potentiation (LTP) was induced using theta-burst stimulation (TBS), consisting of five 100 Hz pulses delivered five times at 200 ms intervals, repeated three times with 20s interval. Responses were normalized to a 10-min pre-TBS baseline.

### Enzyme-linked immunosorbent assay

Brain, blood, and visceral adipose tissue (VAT) were collected post-mortem. Blood was centrifuged at 2000 × g (4 °C), and serum was stored at −20 °C. Brain and VAT samples were snap-frozen and stored at −80 °C.

Protein extraction followed manufacturer-specific protocols. Aβ₄₀/₄₂ levels were measured using Invitrogen kits; all other proteins were assessed using AssayGenie ELISAs (Supplementary Table 3). Approximately 200 μg of tissue was used per homogenate.

For non-Aβ immunoassays, tissues were rinsed in 1 × PBS, minced, and homogenized in PBS (9 mL/g) with protease inhibitors. Lysates were sonicated (60 Hz) and centrifuged at 5000 × g for 5 min at 4 °C. Supernatants were used for ELISA; pellets stored at −80 °C.

Aβ₄₀/₄₂ extraction followed Invitrogen protocol: tissues were homogenized in 5 M guanidine-HCl (8 vol, 50 mM Tris) in 100 μL increments, mixed at room temperature for 4 h with periodic vortexing, diluted 1:10 in cold PBS with inhibitors, and centrifuged at 16,000 × g for 20 min at 4 °C. Supernatants were used for ELISA; pellets stored at −80 °C.

### Cholesterol measurements

Serum samples were analyzed at the MEDIFOS Center of Laboratory Medicine & Molecular Diagnosis for total cholesterol, LDL-C, HDL-C, and triglycerides. Quantification was performed using Indiko™ and Konelab analyzers with the Cholesterol Insert (Thermo Fisher Scientific, REF: 981813). Cholesterol esters were enzymatically hydrolyzed by cholesterol esterase to yield free cholesterol and fatty acids. The free cholesterol was then oxidized by cholesterol oxidase to cholest-4-en-3-one and hydrogen peroxide. The resulting hydrogen peroxide reacted with hydroxybenzoic acid (HBA) and 4-aminoantipyrine (4-AAP) to generate a chromophore (quinoneimine dye), measured spectrophotometrically at 500–550 nm (Allain et al., 1974).

### Lipidomics

Approximately 20 mg of VAT (stored at −80 °C) was sent to the Lipometrix – KU Leuven Lipidomics Core Facility (Katholieke Universiteit Leuven, Belgium) for lipidomic profiling. Using hydrophilic interaction liquid chromatography coupled with tandem mass spectrometry (HILIC LC–MS/MS), over 2000 lipid species spanning 16 lipid classes were quantified and analyzed. Quantification was performed using class-specific deuterated internal standards and corrected for isotopomer overlap. Statistical significance was assessed using one-way ANOVA. For comparisons involving unequal group sizes, false discovery rate (FDR)-adjusted *p*-values were calculated using the Benjamini–Hochberg method (Talebi et al., 2023).

### Bioinformatic analysis of lipidomics data

LipidMaps was used to retrieve LipidMaps IDs for statistically significant lipids (*p* < 0.05) identified under the H₂O, Alirocumab, and Gliclazide conditions (Fahy et al., 2007; Sud et al., 2007). Lipids lacking sufficient annotation were excluded from downstream analyses. Venn diagrams, generated using the Venn tool, were used to visualize shared and unique lipid classes among WT, 5xFAD, 5xFAD + Alirocumab, and 5xFAD + Gliclazide groups.

To identify lipid-associated proteins in *Mus musculus*, significant lipids were cross-referenced with the SwissLipids database (Aimo et al., 2015). Protein identifiers were converted to official symbols and names using UniProt (UniProt Consortium, 2019). Venn diagrams were again used to highlight overlapping and condition-specific proteins.

Pathway enrichment was performed using Metascape (Zhou et al., 2019), a web-based platform that integrates gene annotation, pathway enrichment, protein–protein interaction networks, and functional clustering across 40 curated databases. Venn diagrams were also employed to identify shared and distinct pathways across experimental conditions.

### Enrichment clustering

Functional enrichment was performed using Metascape, which compares input gene lists to curated gene sets based on biological processes, cellular localization, enzymatic activity, and pathway involvement. Statistically overrepresented pathways are identified using a hypergeometric test with Benjamini–Hochberg correction, providing insight into relevant biological mechanisms.

To reduce redundancy, Metascape calculates Kappa similarity scores between enriched terms. Terms are hierarchically clustered, and clusters are defined using a 0.3 similarity threshold. The most statistically significant term within each cluster (lowest *p*-value) is selected to represent the group in summary visualizations (e.g., bar plots, heatmaps). This method improves interpretability by grouping related annotations and minimizing ontological overlap. Enrichment networks are constructed by linking terms (nodes) with Kappa similarity > 0.3. Nodes can be colored by *p*-value or cluster identity. Local clusters form tightly connected subnetworks, with occasional bridging terms linking biologically related processes.

Kappa statistics, used to assess similarity beyond chance agreement, support robust clustering. Log-transformed *p*-values (e.g., −2 = 0.01) are used for scoring. Complete results, including network structure and term clusters, are available in the metascape_results Excel file within the Supplementary zip archive.

Pathway enrichment draws from KEGG, GO Biological Process, GO Molecular Function, and GO Cellular Component databases, with *Mus musculus* selected as the reference organism.

### Protein interaction and complex analysis

Metascape also performs protein network analysis using MCODE, an algorithm designed to identify densely connected subnetworks (protein complexes) within interaction data. Networks are constructed using BioGRID, and supplemented with additional datasets such as InWeb_IM and OmniPath. MCODE extracts highly interconnected protein modules from the input list, and annotates each complex by integrating the top three significantly enriched ontology terms. This allows for functional interpretation of protein complexes within the broader interactome context.

### MCODE algorithm

The Molecular Complex Detection (MCODE) algorithm identifies densely connected regions within large protein–protein interaction (PPI) networks, which may represent functional molecular complexes. The method applies vertex weighting based on local neighborhood density and expands outward from high-density seed proteins to isolate clusters according to defined parameters. Unlike standard graph-based clustering, MCODE offers a directed mode, enabling refined analysis of specific regions without requiring global network traversal.

Predicting molecular complexes enhances functional annotation by inferring coordinated biological roles among interacting proteins. As subunits of a complex typically act toward a common function, assigning uncharacterized proteins to these structures increases confidence in their predicted roles. MCODE also facilitates network visualization by extracting functionally coherent subnetworks.

Gene Ontology (GO) enrichment analysis was applied to each MCODE-derived cluster using Metascape, and the top three terms (based on *p*-value) were retained to describe each module’s biological function. Each MCODE cluster was assigned a distinct color for clarity in network visualization.

### Experimental design and statistical analyses

All experimental procedures were performed in accordance with EU Directive 86/609/EEC and were approved by the institutional animal care committee. A total of 67 male mice were included in the study. Due to mortality-related challenges during the breeding phase, only male offspring were selected to ensure sufficient group sizes and maintain the statistical power of the analysis.

The 5xFAD transgenic mouse line was used as an Alzheimer’s disease (AD) model, with age-matched wild-type (WT) littermates serving as controls. Mice were randomly assigned to six groups: WT (*n* = 9), WT + Gliclazide (*n* = 12), WT + Alirocumab (*n* = 12), 5xFAD (*n* = 10), 5xFAD + Gliclazide (*n* = 12), and 5xFAD + Alirocumab (*n* = 12). No animals were excluded. Treatment began at 3 months of age and continued for 5 months. Alirocumab (10 mg/kg) was administered subcutaneously every 10 days; Gliclazide (20 mg/kg) was given via oral gavage three times per week. Behavioral testing, electrophysiological recordings, ELISA, lipidomics, and cholesterol measurements were performed at 8 months of age.

For electrophysiology, hippocampal slices were collected and field excitatory postsynaptic potentials (fEPSPs) were recorded in the CA1 region after Schaffer collateral stimulation. Long-term potentiation (LTP) was induced using theta-burst stimulation. For molecular analyses, serum and brain tissues were processed for ELISA and immunoassays. Visceral adipose tissue (VAT) was subjected to HILIC LC–MS/MS lipidomic analysis, with statistical comparisons performed via one-way ANOVA and false discovery rate (FDR) correction (Benjamini–Hochberg method).

Normality was assessed using the Shapiro–Wilk test. Group comparisons were analyzed using one-way or two-way ANOVA, followed by Tukey’s *post-hoc* test. Student’s t-test was used for two-group comparisons. Statistical significance was set at *p* ≤ 0.05. Analyses were conducted in GraphPad Prism v8.00 (GraphPad Software, San Diego, CA). Pathway and enrichment analyses were performed in Metascape using the hypergeometric test with FDR correction.

Sample sizes were informed by pilot studies and existing literature using comparable behavioral and electrophysiological endpoints. Although no formal power analysis was performed, group sizes are consistent with those sufficient to detect large effect sizes in transgenic AD models. Data supporting the findings of this study are available from the corresponding author upon reasonable request.

## Results

### Alirocumab restores cognitive function and protects against potential hippocampal atrophy in 5xFAD mice

**Figure 1:**
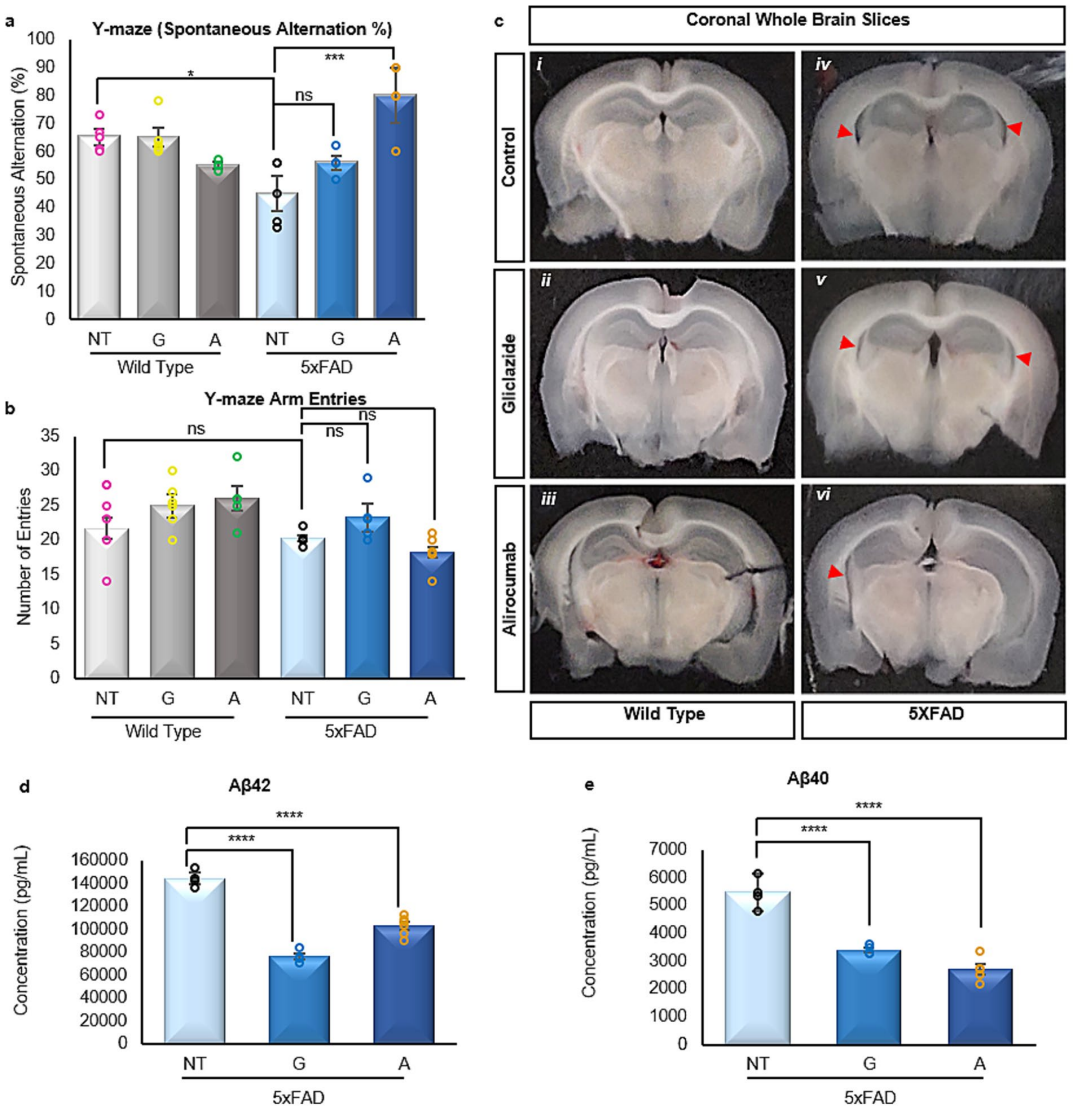
**(a)** Assessment of spatial awareness and cognition via the Y-maze test. The WT mice groups demonstrate an unaffected cognitive capacity, while the 5xFAD group treated with Alirocumab showed a significant reduction in cognitive function [F (5,20)=13.26]. **(b)** Number of mouse entries in the arms of the Y-maze, ensuring proper motor function. **(c)** Representative brain coronal sections from each mouse group. Observed hippocampal atrophy (red arrows) in the 5xFAD groups (iv, v, vi), which is more prominent in the untreated group (iv), and minimal in the Alirocumab group (vi) **(d,e)**. Enzyme linked immunosorbent assay of the two most prominent amyloidogenic species of Aβ in AD, namely Aβ42 [F (2,10)=171.2] **(d)** and Aβ40 [F (2,8)=94.08] **(e)**. Treatment with either Gliclazide or Alirocumab significantly reduced the concentrations of the two prefibrillar amyloidogenic species. Mean ± SEM. One-way ANOVA and Tukey’s *post-hoc* test. ns = not significant, *p ≤ 0.05, **p ≤ 0.01, ***p ≤ 0.001 and ****p ≤ 0.0001.

Cognitive impairment in AD is generally attributed to widespread and progressive neurodegeneration, specifically within the hippocampus. In order to evaluate the impact of Alirocumab and Gliclazide treatment on the animals’ cognitive performance, both the 5xFAD and the WT mice were subjected to the Y-maze spontaneous alternation test in order to assess both spatial working memory and exploratory behavior. Our findings, as depicted in Figure 1a, demonstrate that while the cognitive capacity of WT animals remained unaffected with the administration of either medication, 5xFAD mice treated with Alirocumab exhibited a statistically significant improvement in cognition of up to 78%. On the other hand, 5xFAD mice treated with the anti-glycemic agent Gliclazide did display a modest enhancement in spatial awareness, but these observations failed to attain statistical significance. The number of Y-maze arm entries appears to not change significantly between wild type and 5XFAD animals, irrespective of treatment protocol as shown in Figure 1b, indicative of the absence of any motor defects caused by treatment. Furthermore, WT animals treated with either Gliclazide or Alirocumab did not present any statistical differences in terms of arm entries nor spatial alternation percentage.

Preliminary examination of coronal sections spanning the brain revealed some unexpected evidence of hippocampal atrophy in the 5xFAD animals (Figure 1c iv), albeit to a lesser extent in the Gliclazide-treated 5xFAD animals (Figure 1a iv). Conversely, the observed atrophy in the 5xFAD Alirocumab treated animals was very minimal in comparison (Figure 1c vi). The validity of this preliminary observation will be explored further in future studies where quantification and concise examination can be carried out.

One of the hallmarks of AD is the widespread presence of prefibrillar amyloidogenic species of Αβ, most notably Αβ40 and Αβ42, as previously recorded in the 5xFAD mouse model (Oakley et al., 2006). Our results obtained through enzyme-linked immunosorbent assay indicate that treatment with either Gliclazide or Alirocumab significantly reduces both species (Figures 1d,e).

### Alirocumab restores synaptic plasticity and long-term potentiation in 5xFAD mice

Cognitive function is closely linked to synaptic integrity and plasticity, both of which are progressively impaired in AD. To determine whether Alirocumab and Gliclazide treatment influenced synaptic function in any meaningful way, we conducted a thorough functional examination of the CA3-CA1 synaptic responses and long-term potentiation (LTP). LTP refers to the enduring enhancement of synaptic transmission between two neurons, resulting from the persistent reinforcement of synapses based on recorded and varying activity patterns (Cooke and Bliss, 2006). Importantly, LTP plays a pivotal role in synaptic plasticity, which pertains to the ability of chemical synapses to modify their strength and thus encode memories. Evoked fEPSPs (field excitatory post-synaptic potentials) were recorded in the CA1 region of the hippocampus through acute ex vivo brain slices from the different mouse groups to evaluate their synaptic response to increasing current stimulation and their ability to potentiate the synaptic response following theta-burst stimulation.

**Figure 2:**
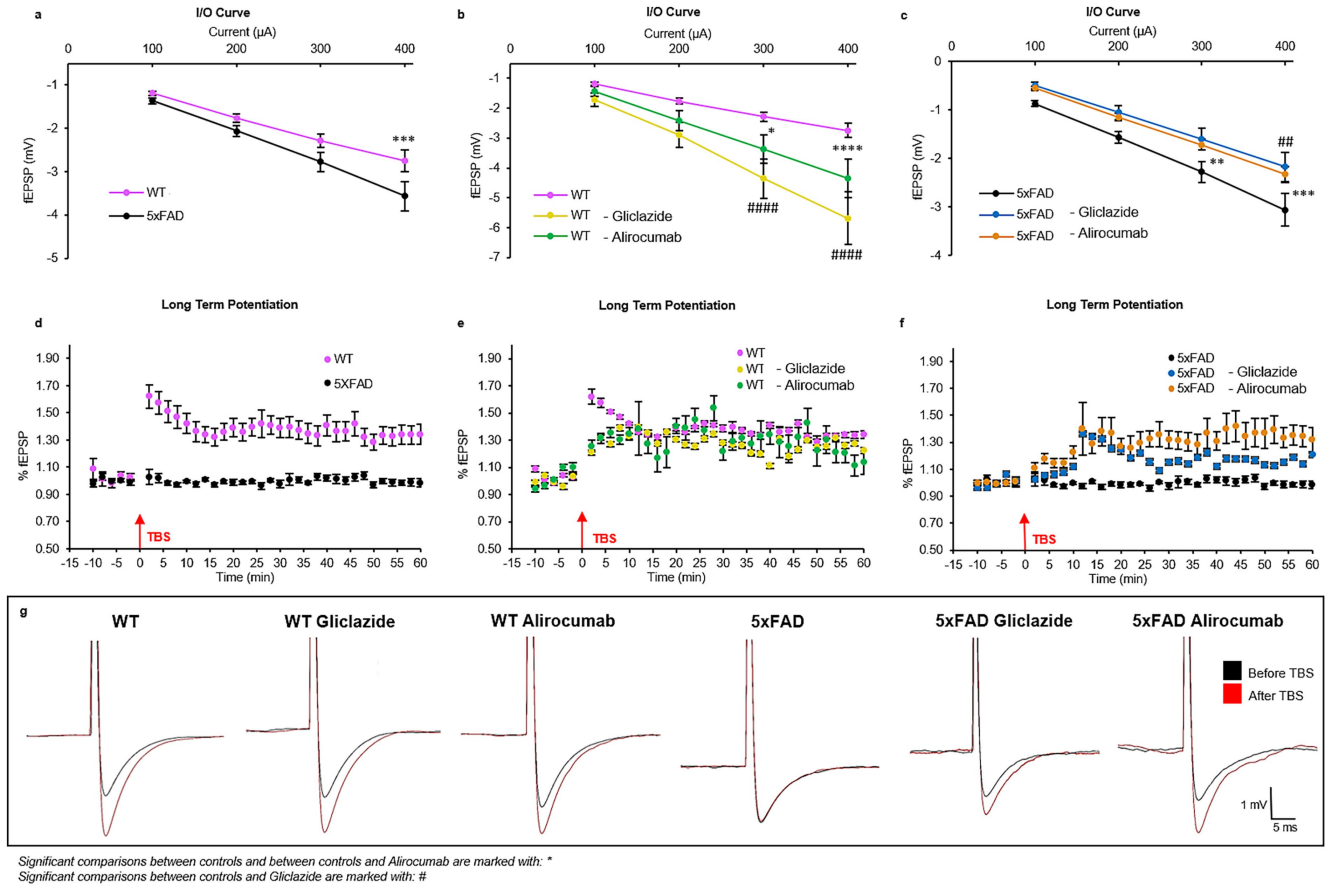
Graphical representation of the electrophysiology results showing long term potentiation (LTP) and synaptic response (input/output response). **(a)** Graph showing the synaptic response of untreated WT mice compared to untreated 5xFAD mice, with the former having a significantly lower synaptic response compared to the latter (*p*-value = 0.0007). **(b)** The synaptic response of the WT Gliclazide and WT Alirocumab mice is significantly increased compared to the WT control group (*p*-value < 0.0001). **(c)** The synaptic response of the 5xFAD Gliclazide and 5xFAD Alirocumab t mice is significantly lower when compared to the 5xFAD control group (*p*-value = 0.0059 and 0.0004, respectively). **(d)** LTP of WT (pink) and 5xFAD (black) untreated mice. The 5xFAD control mice showed an absence of LTP, whereas WT control mice had the typical curve form (*F*(75,598), *p*-value < 0.0001). **(e)** Comparison of LTP between the WT control, WT Gliclazide (yellow) and WT Alirocumab (green) mice (*F*(75,675) = 40.39, *F*(75,599) = 20.21, respectively). No significant difference between any of the three groups, indicating that the drugs do not affect the LTP in WT mice. **(f)** Comparison of LTP between 5xFAD control, 5xFAD Gliclazide (blue) and 5xFAD Alirocumab (orange) mice. 5xFAD- Alirocumab display a significant increase of the LTP (*F*(75,672), *p*-value < 0.0001). Although 5xFAD- Gliclazide group showed an enhanced response, no statistical significance was obtained (*F*(75,523) = 26.68). **(g)** Representative traces before (black) and after (red) theta-burst stimulation. Mean ± SEM. One-way and Two-way ANOVA and Bonferroni’s *post-hoc* test.

Compared to untreated wild-type (WT) mice, untreated 5xFAD mice exhibited a significantly enhanced synaptic response, as indicated by the input–output (I/O) curve (Figure 2a). However, treatment with either Gliclazide or Alirocumab in 5xFAD mice resulted in a marked reduction in synaptic responses relative to untreated 5xFAD controls (Figure 2c). On the contrary, the synaptic responses of WT mice treated with both medications were found to be higher than the untreated WT group, however when compared to each other no statistical significance was recorded (Figure 2b).

Concerning the LTP, untreated WT mice exhibited the expected increase in synaptic response following the theta burst (pink dots), while the untreated 5xFAD mice exhibited a flat response, indicating the absence of LTP (black dots) (Figure 2d). Regarding the effect of the different drugs on LTP, no significant difference was observed among the different treatment groups of WT mice (Figure 2e). In contrast, 5xFAD mice treated with Alirocumab exhibited a statistically significant higher LTP than the control group of 5xFAD mice, comparable to the values obtained with the WT, while 5xFAD mice treated with Gliclazide showed a trend for but not a statistically significant increase in the LTP compared to control 5xFAD (Figure 2f). Detailed, point-point values can be found in Supplementary Table 4. The traces shown in Figure 2g represent the amplitude of the fESPS at a normal condition (black). After theta burst stimulation (TBS) the traces show the new amplitude representing the strengthening/enhancement upon reaching LTP state. WT control mice show great increase in the fEPSP amplitude after TBS, however 5xFAD control mice show no difference at all meaning that no LTP occur. Post treatment the amplitude rises again to show the successful enhancement state (LTP).

### Gliclazide and Alirocumab influence neuronal and glial cell populations

**Figure 3:**
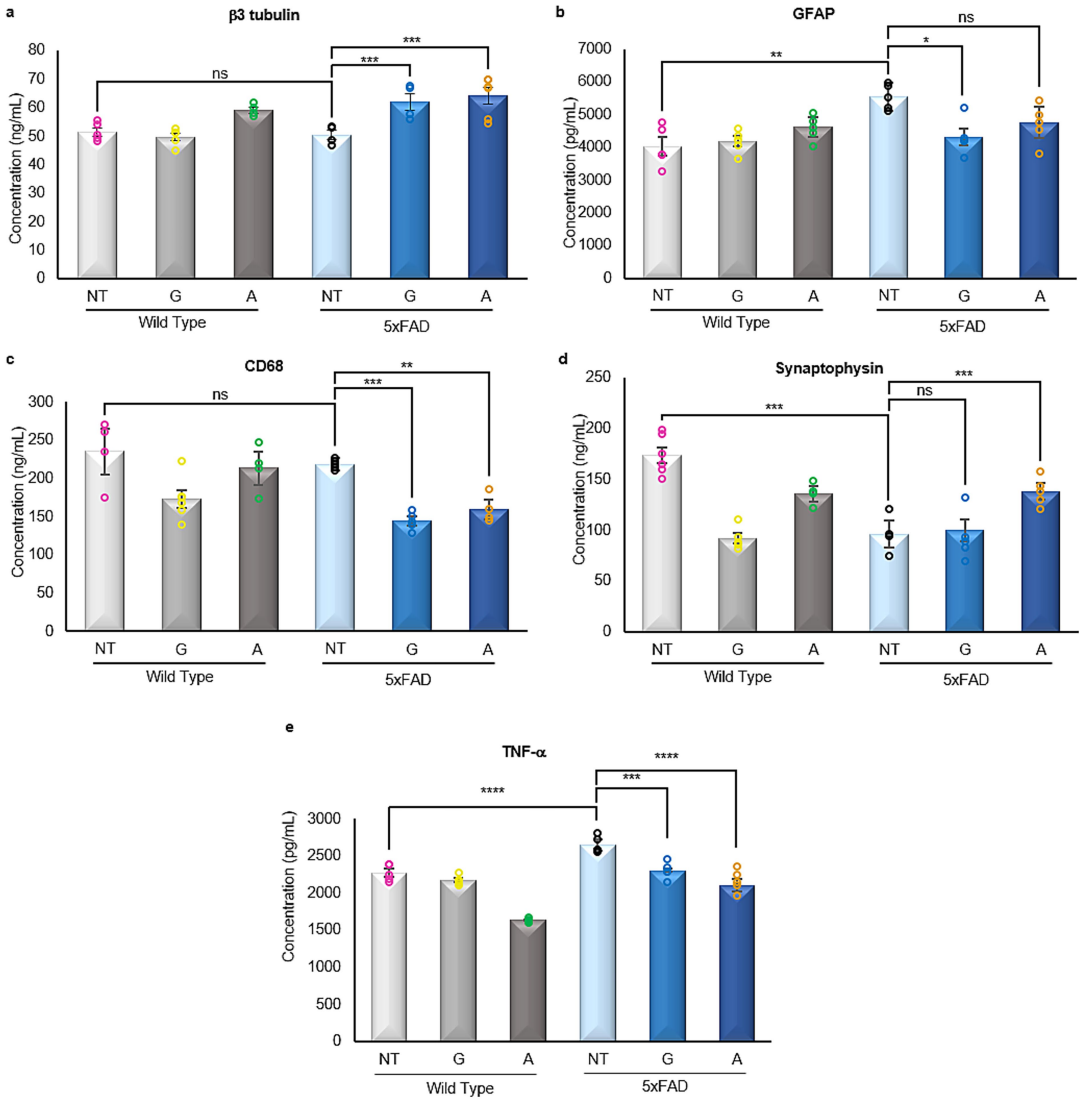
Enzyme linked immunosorbent assays of neural markers. **(a)** The pan-neuronal marker β3 tubulin is not affected by any treatment in the WT mice groups, however, there is a significant increase in its expression in the 5xFAD-treated mice [F (5,25)=18.88]. **(b)** The astrogliosis marker GFAP is significantly increased in the 5xFAD untreated mice when compared to WT untreated mice. The 5xFAD-Gliclazide mice showed a significant reduction of astrogliosis when compared to the 5xFAD untreated mice, whereas the 5xFAD-Alirocumab mice exhibited no statistical difference [F (5,20)=5.425]. **(c)** The expression of pan-macrophage marker CD68 was significantly reduced in 5xFAD mice following both treatments [F (5,25)=15.52]. **(d)** The expression of synaptophysin is significantly reduced in the 5xFAD-untreated and 5xFAD-Gliclazide groups when compared to the WT-untreated mice. However, following treatment with Alirocumab, a significant increase in synaptophysin was observed [F (5,25)=31.96]. **(e)** The expression of TNF-*α* in the 5xFAD untreated mice was significantly increased when compared to the untreated WT group. After administration with either drug, the TNF-α levels were significantly reduced [F (5,24)=50.98]. Mean ± SEM. One-way ANOVA and Tukey’s *post-hoc* test. ns, not significant, *p ≤ 0.05, **p ≤ 0.01, ***p ≤ 0.001 and ****p ≤ 0.0001.

Given the progressive cognitive decline in AD, neuronal, astrocytic, and microglial populations undergo substantial pathological alterations. To investigate these changes in relation to the observed cognitive improvements, expression levels of representative markers for neurons, astrocytes, and microglia were assessed in brain tissue (Figures 3a–c).

Immunoassay analysis revealed no significant changes in neuronal marker expression between WT and untreated 5xFAD mice. However, treatment with either Gliclazide or Alirocumab resulted in increased expression of the pan-neuronal marker b3 Tubulin (Figure 3a). As expected, astrocytic GFAP expression was elevated in untreated 5xFAD mice, consistent with AD-associated gliosis. Notably, Gliclazide treatment significantly reduced GFAP levels (Figure 3b). Similarly, expression of the pan-macrophage marker was markedly decreased in 5xFAD mice following both treatments (Figure 3c).

### Alirocumab enhances synaptic integrity in 5xFAD mice

Synaptic loss is considered one of the main hallmarks of AD where the progressive accumulation of Αβ plaques and neurofibrillary tangles eventually leads to the degeneration and loss of synapses. This gradual synaptic loss eventually leads to the cognitive impairment observed in AD.

Our analysis also corroborates the expected synaptic loss when comparing the expression levels of synaptophysin in untreated 5xFAD animals compared to their WT control untreated group (Figure 3d). In addition, while no change was recorded in 5xFAD animals treated with Gliclazide, the group of 5xFAD animals treated with Alirocumab displayed a significant increase in synaptophysin expression when compared to the untreated 5xFAD animals (Figure 3d).

### Gliclazide and Alirocumab and decrease neuroinflammation in 5xFAD mice

Neuroinflammation in AD is driven, in part, by elevated pro-inflammatory cytokines such as TNF-*α*, which contribute to neuronal dysfunction and disease progression.

Our analysis revealed a significant increase in TNF-α expression in untreated 5xFAD mice compared to WT controls (Figure 3e). Treatment with either Gliclazide or Alirocumab markedly reduced TNF-α levels in 5xFAD mice, indicating suppression of the inflammatory response. Interestingly, a reduction in TNF-α expression was also observed in WT animals treated with Alirocumab alone.

### Alirocumab restores leptin and omentin expression in 5xFAD mice

Adipokines represent a class of hormones central to the regulation of metabolism and inflammation, originating primarily from the adipose tissue. Notably, these adipokines have demonstrated noteworthy influence extending beyond metabolic processes, exerting considerable effects on the brain and the comprehensive functioning of the central nervous system. Research has indicated that adipokines exert a significant influence on both glucose metabolism and insulin signaling (Morigny et al., 2021; Al-Mansoori et al., 2022), two intricate processes profoundly implicated in AD that affect both neuronal function and survival.

Given the substantial importance of these hormones in the progression and development of AD, immunoassays were conducted to examine the expression of prominent adipokines within the brains of both the WT control groups and 5xFAD mice, treated and untreated. Due to the small amount of sample, only the three main adipokines were also quantified in serum.

**Figure 4:**
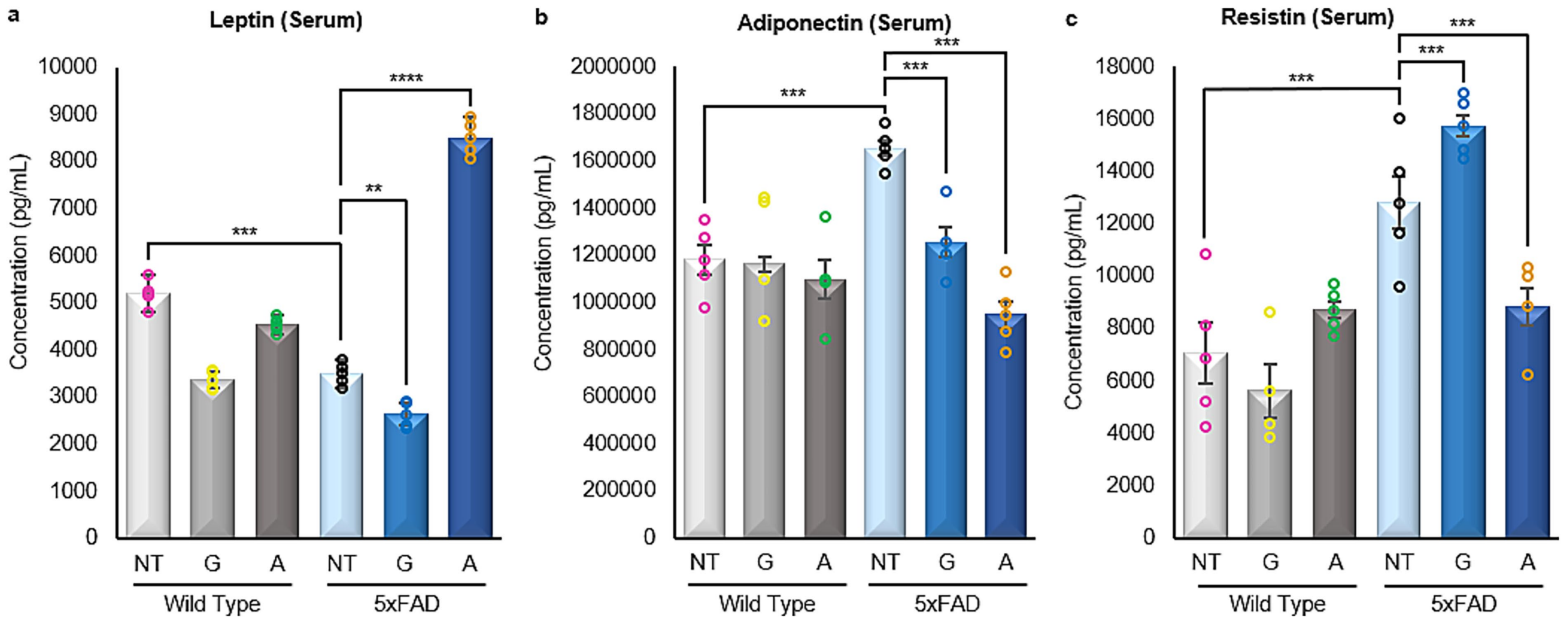
Enzyme linked immunosorbent assays of adipokines in serum samples. **(a)** A significant decrease in the levels of Leptin was observed in the untreated 5xFAD mice cases when compared with the WT untreated mice. Significant increase was only observed following the treatment of the 5xFAD animals with Alirocumab [F (5,24)=345,3]. **(b)** The level of Adiponectin expression was drastically increased in 5xFAD animals when compared to their WT counterparts. Adiponectin levels however returned to the expected values when the 5xFAD animals were treated with Alirocumab [F (5,22)=9.394]. **(c)** The levels of Resistin were found increased in 5xFAD animals when compared to the WT animals. Resistin levels were further increased following treatment of the 5xFAD animals with Gliclazide but again returned to physiological levels following Alirocumab treatment [F (5,22)=18.23]. Mean ± SEM. One-way ANOVA and Tukey’s *post-hoc* test. ns, not significant, *p ≤ 0.05, **p ≤ 0.01, ***p ≤ 0.001 and ****p ≤ 0.0001.

Analysis of serum samples from all mice included in the study showed that untreated 5xFAD animals had statistically lower levels of Leptin and higher levels of Adiponectin when compared to their WT counterparts. However, 5xFAD mice treated with Alirocumab showed a statistically significant increase in the expression of Leptin in the serum (Figure 4a), and a marked decrease in the expression of Adiponectin (Figure 4b). Similarly, the levels of Resistin in the serum which was found to be significantly increased in the untreated 5xFAD animals were decreased following treatment of 5xFAD animals with Alirocumab (Figure 4c).

**Figure 5:**
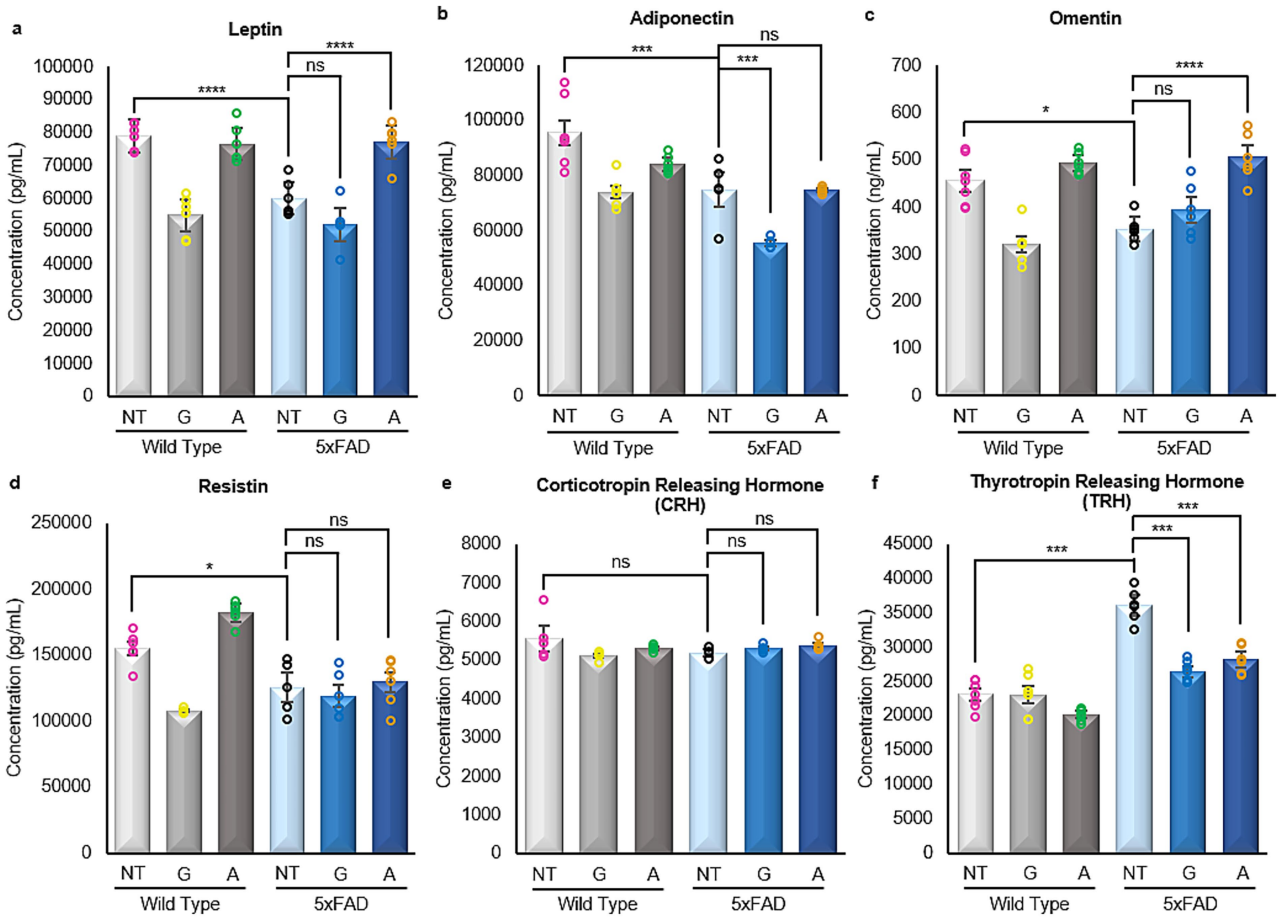
Enzyme linked immunosorbent assays of adipokines and hormones in brain samples. **(a–d)** All four adipokines, namely Leptin [F (5,30)=35.98], Adiponectin [F (5,30)=25.61], Omentin [F (5,25)=20.10] and Resistin [F (5,25)=21.32] showed very similar results. A significant decrease was observed in the untreated 5xFAD mice in all cases when compared with the WT untreated mice. Following Gliclazide treatment, only adiponectin was found to be significantly reduced. On the contrary, a significant increase in leptin and omentin levels was noted in 5xFAD mice after Alirocumab administration. **(e)** No significant changes were found in the expression of CRH [F (5,25)=1.075]. **(f)** The expression of TRH was significantly increased in the untreated 5xFAD mice when compared with their corresponding WT group. However, significantly decreased expression of TRH was observed in mice treated with Gliclazide and Alirocumab [F (5,25)=69.50]. Mean ± SEM. One-way ANOVA and Tukey’s *post–hoc* test. ns, not significant, *p ≤ 0.05, **p ≤ 0.01, ***p ≤ 0.001 and ****p ≤ 0.0001.

All four adipokines were decreased in the brain tissue of 5xFAD mice compared to wild-type (WT) controls (Figures 5a–d). Gliclazide treatment led to a significant reduction in adiponectin levels but had no effect on leptin or omentin (Figure 5b, *p*-value = 0.0002). In contrast, Alirocumab significantly increased leptin and omentin levels but did not alter adiponectin concentrations in 5xFAD brain tissue (Figure 5a, *p*-value<0.0001 and 5c, *p*-value<0.0001, respectively). Resistin levels remained unchanged following treatment with either compound.

### Gliclazide and Alirocumab restore thyrotropin-releasing hormone expression to basal levels in 5xFAD animals

Following their release from adipose tissue, adipokines cross the blood–brain barrier and bind to receptors in the hypothalamus, modulating key physiological processes such as appetite regulation, metabolic homeostasis, insulin sensitivity, lipid and glucose metabolism, endothelial function, blood pressure, hemostasis, neuroendocrine signaling, and immune responses.

As previously mentioned, immunoassays were carried out on all the extracted brain tissues obtained from all the animals included in the study, both WT and 5xFAD, treated and untreated to examine two specific hormones secreted by the hypothalamus in reaction to adipokine stimulus within the context of this study. No changes were found in the expression of corticotropin-releasing hormone (CRH; Figure 5f), a hormone known to regulate both glucose and lipid metabolism through stimulation of glucose release from the liver and lipolysis, respectively. Nevertheless, significant changes were observed in the expression of thyrotropin-releasing hormone (TRH) (Figure 5e). Specifically, untreated 5xFAD mice exhibited a significant increase in TRH expression compared to their WT counterparts. Nevertheless, treatment with Gliclazide and Alirocumab resulted in a prominent decrease in TRH expression, effectively restoring it to levels observed in untreated WT animals.

### Gliclazide and Alirocumab restore insulin levels in 5xFAD mice

Insulin signaling plays a critical role in brain metabolism, and its dysregulation has been implicated in AD pathology. Insulin-degrading enzyme (IDE), a key regulator of insulin clearance and amyloid-*β* catabolism, is also frequently disrupted in AD.

**Figure 6:**
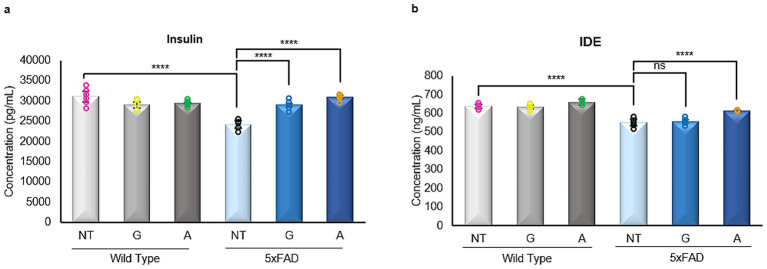
Enzyme linked immunosorbent assays of insulin and insulin degrading enzyme (IDE). **(a)** A significant decrease is observed in the 5xFAD untreated mice, when compared to the WT untreated group. After administration with either drug, 5xFAD mice groups showed a significant increase in Insulin expression [F (5,26)=17.96]. **(b)** Similarly, there is a significant decrease of the IDE expression in the 5xFAD untreated mice when compared to their WT untreated littermate. No significant change is to be observed after Gliclazide administration, however, administration of Alirocumab increased significantly the expression of IDE when compared to the 5xFAD controls [F (5,25)=40.44]. Mean ± SEM. One-way ANOVA and Tukey’s *post-hoc* test. ns, not significant, *p ≤ 0.05, **p ≤ 0.01, ***p ≤ 0.001 and ****p ≤ 0.0001.

In our study, untreated 5xFAD mice exhibited a significant reduction in both insulin and IDE levels compared to WT controls (Figures 6a,b). Treatment with either Gliclazide or Alirocumab restored insulin levels in 5xFAD animals to those observed in WT mice (Figure 6a). However, only Alirocumab treatment led to a significant increase in IDE expression in 5xFAD mice, while Gliclazide-treated animals showed no significant change compared to the untreated 5xFAD group (Figure 6b).

### Alirocumab restores LDL cholesterol to wild-type levels in 5xFAD mice

**Figure 7:**
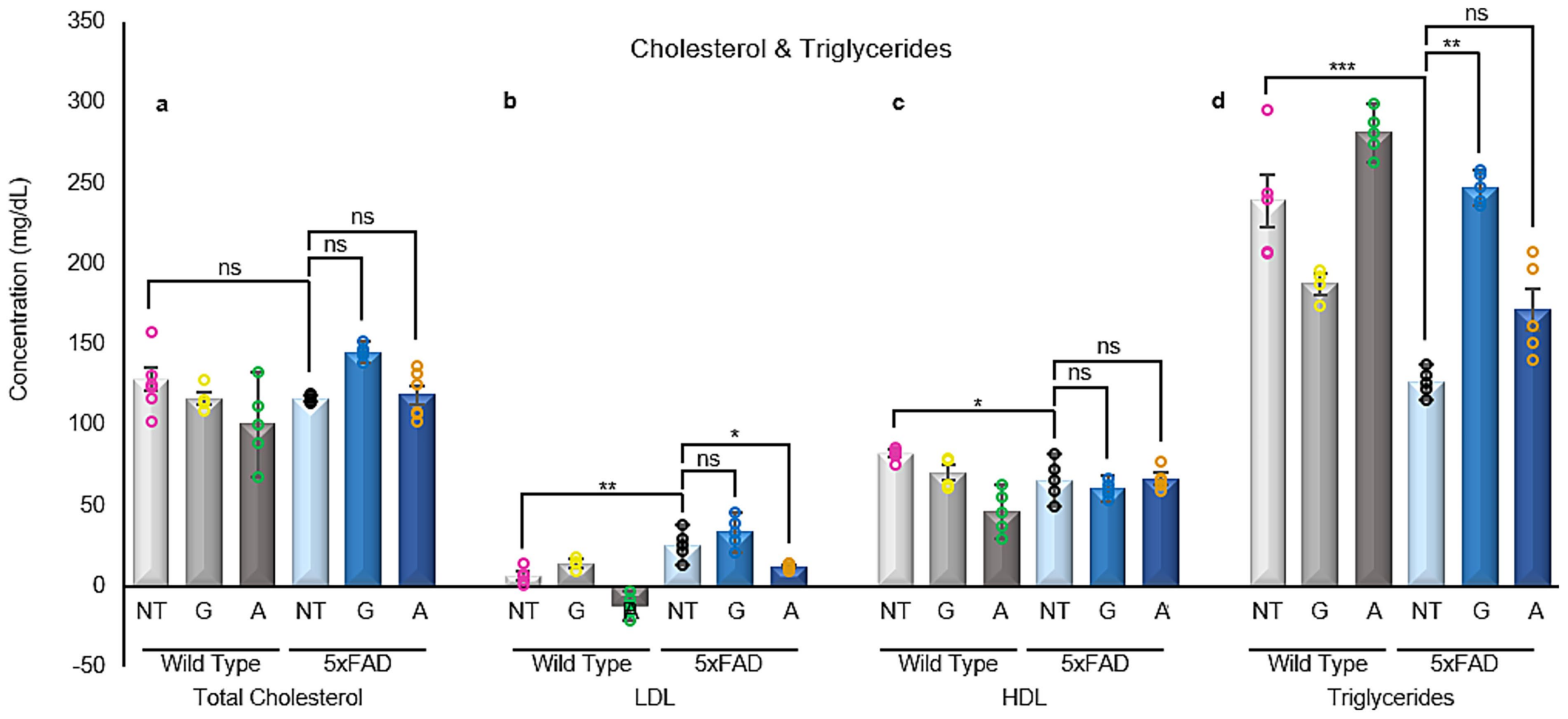
Cholesterol measurement in blood serum samples. **(a)** Total cholesterol remain unchanged in their entirety among all groups. **(b)** Significant changes can be seen in LDL-cholesterol levels when comparing untreated 5xFAD mice with their untreated WT littermates. Post Alirocumab treatment, 5xFAD mice exhibited a significant decrease in the LDL expression levels, restoring them to the levels recorded in WT mice. **(c)** HDL-cholesterol levels are found to be significantly reduced in the untreated 5xFAD mice when compared to the untreated WT group. However, no significant change was observed after administration of either drug. **(d)** Triglycerides levels were significantly reduced in the 5xFAD untreated mice when compared to their WT littermates. Administration of Gliclazide restore their levels to normal values. Mean ± SEM. One-way and Two-way ANOVA and Tukey’s *post-hoc* test. ns, not significant, *p ≤ 0.05, **p ≤ 0.01, ***p ≤ 0.001 and ****p ≤ 0.0001. Total cholesterol: F (5,13)=1.802, LDL: F (5,11)=14.17, HDL: F (5,11)=4.721 and Triglycerides F (5,12)=15.01.

Given the cognitive improvements observed in 5xFAD mice following Alirocumab treatment, brain cholesterol levels were assessed across all groups. The analysis revealed that total and HDL cholesterol levels remained unchanged regardless of genotype or treatment (Figure 7). However, both untreated and Gliclazide-treated 5xFAD mice showed significantly elevated LDL levels compared to WT controls. Notably, Alirocumab-treated 5xFAD mice exhibited a marked reduction in brain LDL levels, comparable to those of WT mice (Figure 7).

### Gliclazide and Alirocumab reshape fatty acid composition in visceral adipose tissue

Given the detected variations in adipokines found within the brain tissues examined (Figure 5), it is crucial to also undertake the analysis of the composition of the visceral adipose tissue responsible for secreting the adipokines under investigation.

**Figure 8:**
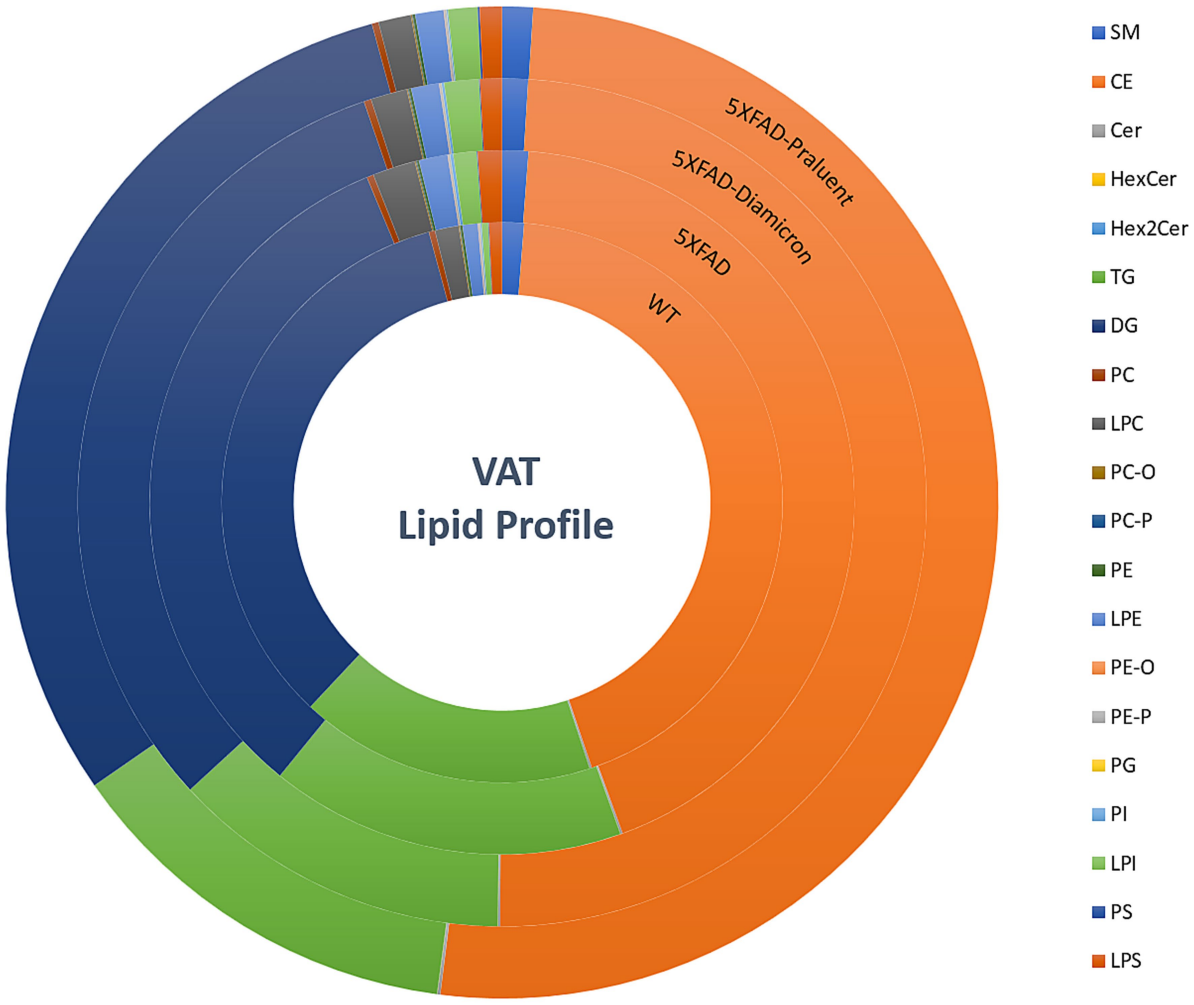
Lipidome profile of visceral adipose tissue. Concentric circles representing the 4 animal groups, starting with untreated WT mice (inner circle), 5xFAD-untreated, 5xFAD- Gliclazide and 5xFAD- Alirocumab (outer circle). Each color represents the percentage (%) of each major lipid class. The lipidomic analysis showed the phospholipid superfamily to be most affected especially regarding the LPE, LPS and LPC subclasses.

Generally, when discussing the lipidomics profile of adipose tissue we refer to the fatty acid composition and abundance. This varying composition essentially indicates the functionality and signaling capability of the tissue as observed from the lipidomic characteristics recorded at the time of analysis. We have carried out untargeted lipidomics on visceral adipose tissue samples from all animals partaking in the study. Our findings reveal distinct profiles when comparing the animals belonging to the WT population to those of the 5xFAD group. Moreover, notable disparities also surfaced among the 5xFAD animals subsequent to their treatment with either Gliclazide or Alirocumab (Figure 8). The full composition list can be found in Supplementary Table 2, including the treated WT animals. The most affected lipid species appear to belong to the phospholipid superfamily, and more specifically referring to the Lysophosphatidylethanolamine (LPE), Lysohosphatidylserine (LPS) and Lysophosphatidylcholine (LPC) subclasses.

Following lipidomics analysis, significantly altered lipids (based on fold change) were associated to interacting proteins (MetaScape). The resulting Venn diagram provided lipids/proteins exclusively found in each of the treatment groups (Supplementary Figure 1). Considering all previously obtained results, we chose to concentrate on the Alirocumab treated FAD mice. Based on existing literature we thus decided to concentrate on the expression of LPIN1 and DGAT1 in our animal brain samples. LPIN1 is known to be involved in both lipid synthesis and storage, as well as the metabolism of several fatty acids. LPIN1 also encodes for phosphatidate phosphatase, an enzyme catalyzing the conversion of phosphatidate to diglyceride, a process ultimately crucial for the production of triglyceride in white adipose tissue. In general, LIPN1 plays a key role in many metabolic syndrome diseases including lipodystrophy, fatty liver, hypertriglyceridemia and insulin resistance (Csaki et al., 2014). In terms of AD, LIPIN1 deficiency has been shown to cause a decrease in the number of hippocampal synapses thus affecting spatial learning and memory (Shang et al., 2020). DGAT1 on the other hand is a triglyceride-synthesizing enzyme catalyzing the conversion of diacylglycerol and fatty acyl CoA to triacylglycerol. It shields the endoplasmic reticulum against lipotoxic stress incurred by events such as adipose tissue inflammation (Chitraju et al., 2017).

**Figure 9:**
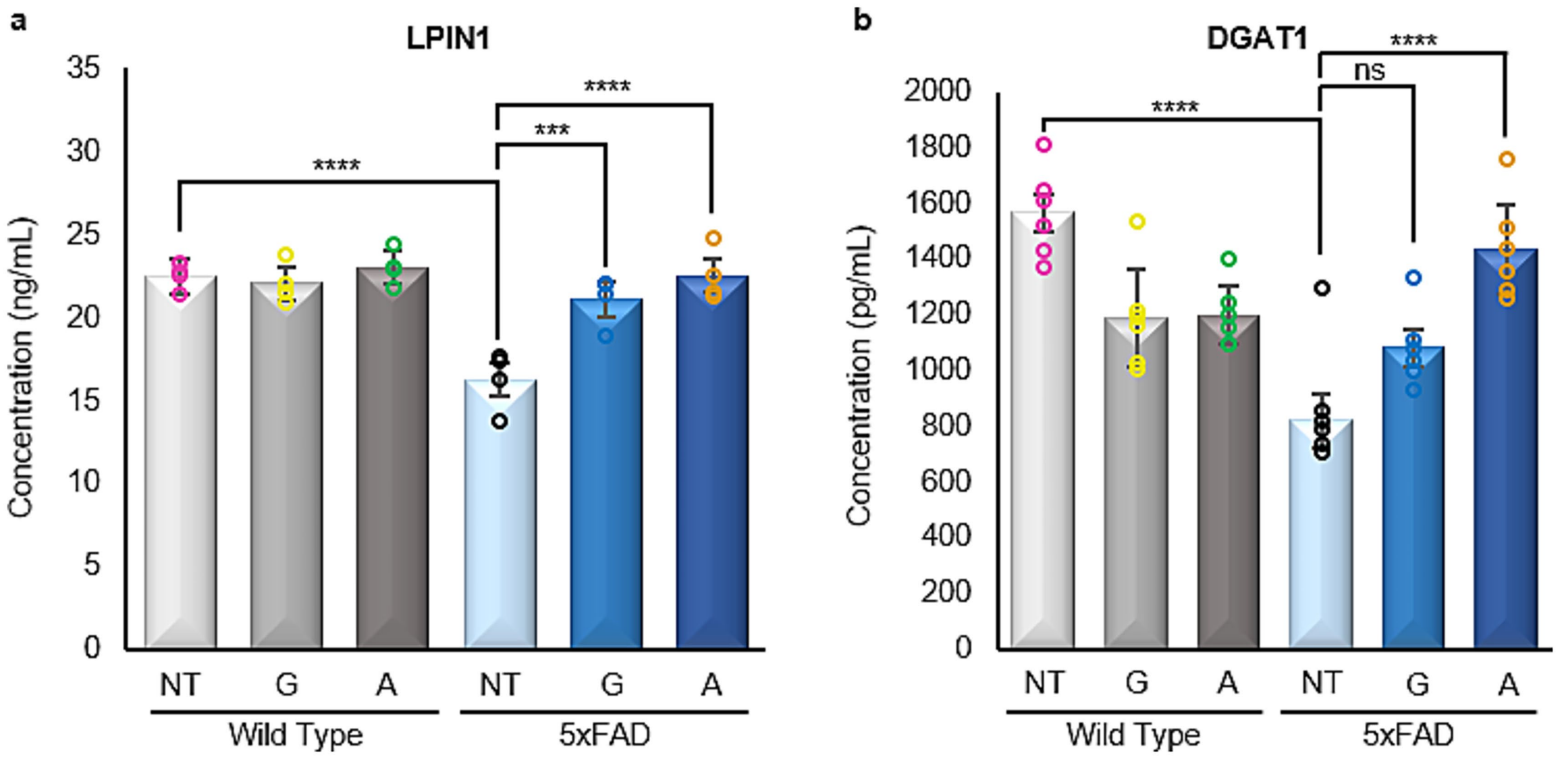
Enzyme linked immunosorbent assays of LIPIN1 and DGAT1. **(a)** A significant decrease is observed in the 5xFAD untreated mice, when compared to the WT untreated group. After administration with either drug, 5xFAD mice groups showed a significant increase in LPIN1 expression [F (5,18)=13.84]. **(b)** Similarly, there is a significant decrease in the DGAT1 expression in the 5xFAD untreated mice when compared to their WT untreated littermate. Administration of Alirocumab increased significantly the expression of DGAT1 when compared to the 5xFAD controls. However, no significant change is to be observed after Gliclazide administration [F (5,30)=18.30]. Mean ± SEM. One-way ANOVA and Tukey’s *post–hoc* test. ns = not significant, *p ≤ 0.05, **p ≤ 0.01, ***p ≤ 0.001 and ****p ≤ 0.0001.

Therefore, we carried out immunoassays concerning these two proteins in all brain samples partaking in this study. 5xFAD untreated mice were found to have a significantly reduced expression of LIPIN1 when compared to their WT counterparts. However, 5xFAD animals treated with either Gliclazide or Alirocumab showed increased expression reaching the level of WT animals (Figure 9a). DGAT1 was also found to be decreased in 5xFAD untreated mice compared to their WT counterparts, whereas 5xFAD animals treated with Alirocumab showed significantly higher expression reaching the level of the WT group (Figure 9b).

## Discussion

AD is one of the most complex and devastating neurodegenerative disorders. There are no treatment options that can offer a sustainable or translatable cure regimen (Cummings et al., 2019; Vaz and Silvestre, 2020). These challenges are direct result of the intricate and multifactorial nature of AD which lie at the very center of the disease’s pathophysiology. This pathophysiology extends far beyond the well-characterized amyloid and tau pathology and has been shown to include not only metabolic dysfunction and neuroinflammation but also widespread synaptic loss which is often accompanied by neuronal loss as AD progresses (Tönnies and Trushina, 2017). Therapeutic efforts have mainly focused on amyloid-reducing or targeting strategies. Emerging research however suggests that both metabolic dysfunction and lipid dysregulation may indeed be central to the disease’s pathophysiology and ultimate progression (Yin, 2023; de la Monte and Tong, 2014; Chew et al., 2020). In this study, we have explored how Alirocumab, a PCSK9 inhibitor, and Gliclazide, an anti-glycemic agent, can impact the disease’s progression in 5xFAD mice, a commonly used murine model of the disease. Our results clearly demonstrate how early metabolic interventions can indeed exert a significant neuroprotective effect while providing compelling evidence that AD pathology is deeply linked to systemic metabolic health and homeostasis.

A particularly prominent outcome of our study was the significant cognitive improvement observed in the Alirocumab-treated 5xFAD group. Our behavioral assessment, through the spatial alternation Y-maze test, revealed a 78% improvement in cognition (Chen et al., 2018). Gliclazide is known to inhibits KATP channels, which are expressed in multiple organs, including the brain. This inhibition leads to membrane depolarization and increased neuronal excitability, thereby enhancing synaptic responses. Alirocumab, by lowering LDL cholesterol levels, may exert neuroprotective effects that support synaptic integrity. Consequently, administration of either drug in wild-type (WT) mice is expected to enhance synaptic activity. In 5xFAD mice, enhanced synaptic responses are also observed, likely reflecting a compensatory mechanism in response to synaptic degeneration. Studies by Tang et al. (in mice) and Huijbers et al. (in humans) report elevated neuronal activity in the hippocampus and other brain regions in both 5xFAD models and individuals with cognitive impairment (Huijbers et al., 2015; Tang et al., 2016). These findings support the hypothesis that compensatory hyperactivity emerges early in Alzheimer’s disease (AD) to preserve cognitive function. Tang et al. specifically noted increased neuronal activity beginning at 2 months of age in 5xFAD mice, persisting through the 5-month experimental period. Based on our data at 9 months, this elevated synaptic activity appears sustained. However, treatment of 5xFAD mice with both Gliclazide and Alirocumab reduces synaptic responses to levels comparable to WT controls. Enhanced LTP and synaptic plasticity measurements recorded via electrophysiology raise another important question. Can Alirocumab act directly on the brain? Or could the observed cognitive benefits instead be an indirect consequence of the improved systemic lipid homeostasis that occurs following Alirocumab administration? Alirocumab’s ability to lower LDL cholesterol has been well documented in cardiovascular research (Gaudet et al., 2022), but its potential use or impact on the central nervous system requires further research. While PCSK9 inhibitors have been implicated in modulating brain lipid metabolism, there is still uncertainty regarding their ability to cross the blood–brain barrier. Further studies examining Alirocumab’s penetration into the central nervous system and its direct influence on neuronal lipid regulation will be critical in resolving this question.

Even if Alirocumab’s effects are largely systemic however, its potential relevance and usefulness in AD therapy still remains strong. Epidemiological studies have consistently linked elevated LDL cholesterol levels to an increased risk of developing AD later in life. This suggests that lipid dysregulation itself may indeed accelerate cognitive decline (Yin, 2023; Akyol et al., 2021). In this study, the observed impact following Alirocumab administration on the synaptic markers synaptophysin and β3 tubulin could indicate that correcting systemic lipid imbalances enhances synaptic resilience and by extension delays the neurodegenerative cascade that underlies AD pathology and progression.

Unlike Alirocumab, Gliclazide treatment did not present with significant cognitive improved cognition in the 5xFAD mice within the timeframe of our study. Gliclazide however did reduce amyloid burden and neuroinflammation which suggests that the primary effects of the medication may instead be preventative rather than restorative (Chen et al., 2011; Rojas et al., 2021). This is an important distinction that exhibits that therapies regulating metabolic pathways may be more effective when administered early in order to precede irreversible synaptic dysfunction. Nevertheless, the reduced recorded cognitive improvement observed with Gliclazide does not necessarily negate its potential therapeutic value in the context of AD. T2D has been a well-established risk factor for AD (Ott et al., 1999; Ott et al., 1996). Gliclazide, a sulfonylurea class antidiabetic agent, primarily functions by stimulating insulin secretion. Chronic administration has been associated with improved glycemic control and, in some cases, modest reductions in adiposity. Given that leptin secretion is closely linked to adipose tissue mass, such reductions may lead to decreased circulating leptin levels (Friedman, 2011). Additionally, Gliclazide exhibits antioxidant properties, which may contribute to its metabolic effects (Choi and Ho, 2018). However, direct evidence linking Gliclazide’s antioxidant properties to reduced leptin levels, independent of insulin regulation, remains limited and warrants further investigation.

Also, particular anti-diabetic medications have previously been associated with an overall reduction in the risk of developing AD (Poor et al., 2021). The fact that Gliclazide can effectively modulate insulin signaling and neuroinflammation suggests that such treatments may instead be more effective in the form of preventative therapies rather than a late-stage intervention. Future studies could instead target pre-symptomatic administration of Gliclazide in AD high-risk populations which would aid in determining whether early and sustained administration could delay disease onset.

A particularly intriguing aspect of our study is the observed relationship between lipid metabolism, adipokines, and synaptic function. While peripheral metabolic dysfunction has long been linked to AD, direct evidence of lipid-driven adipokine changes in the brain remains scarce (Chen and Schneeberger, 2024). Our results show that Alirocumab treatment increased leptin and omentin levels, while adiponectin remained elevated in untreated 5xFAD mice.

Leptin, specifically, has garnered great attention as a potential neuroprotective factor in AD. Leptin receptors are highly expressed in the hippocampus, where leptin signaling is known to enhance synaptic plasticity and modulate tau phosphorylation (Marwarha and Ghribi, 2012; Hamilton and Harvey, 2021). The restoration of expression levels to baseline WT-observed values of leptin and omentin observed in our group of Alirocumab-treated mice may point to a previously unrecognized pathway linking lipid metabolism to synaptic recovery or resilience. However, causality still remains unclear so further studies that employ leptin receptor antagonists or even direct leptin supplementation could be essential in determining whether these adipokine shifts are actively mediating Alirocumab’s significant and rescuing cognitive effects.

Lipidomics analysis provided even further insight into the implication of the lipid metabolism in the pathophysiology of AD. Both Alirocumab and Gliclazide induced significant changes in lipid profiles (Figure 8), however we identified two key regulatory proteins, LPIN1 and DGAT1. Both proteins have a greater implication in regards to lipid synthesis, storage, and fatty acid metabolism, pathways greatly involved with general lipid homeostasis (Shang et al., 2020; Page et al., 2009).

Our findings add to the growing body of evidence that Alzheimer’s is, at least partly, a metabolic disorder of the brain. Lipid dysregulation, insulin resistance, and neuroinflammation not only contribute to the disease’s pathology but may also accelerate cognitive decline. While amyloid deposition remains a defining hallmark of AD, it has become increasingly apparent that targeting solely this feature is ineffective and instead addressing systemic metabolic pathways could open new therapeutic avenues.

The differential effects observed with Alirocumab and Gliclazide highlight the importance of timing, mechanism, and disease stage in determining therapeutic success. Alirocumab’s ability to restore cognitive function suggests a potential for disease modification, whereas Gliclazide’s anti-inflammatory effects may instead be more relevant for early intervention strategies aimed at slowing progression before irreversible synaptic and neuronal loss occurs. From a translational perspective, the fact that Alirocumab is already FDA-approved for hyperlipidemia presents an opportunity for rapid clinical investigation. It would be possible to carry out epidemiological studies targeted at examining cognitive deterioration or improvement in individuals already taking PCSK9 inhibitors thus offering real-world insight into potential neuroprotective effects of the long-term use of these inhibitors. Furthermore, clinical trials designed to assess lipid-lowering therapy in populations with AD high-risk individuals would help identify whether modulating lipid metabolism could serve as an effective early and preventive strategy against the onset of AD.

This study highlights the therapeutic promise of metabolic interventions in AD, demonstrating that targeting lipid and glucose metabolism can influence disease onset and progression. Despite limitations, including sex-restricted animal models, short treatment duration, and uncertain blood–brain barrier penetration of Alirocumab, our findings suggest adipokines are key mediators linking systemic metabolism to synaptic integrity. Possible hippocampal atrophy underscores the need for integrated neuroimaging. Importantly, the results advocate for a shift beyond amyloid-centric strategies, proposing that lipid-modulating therapies like PCSK9 inhibitors may offer scalable, disease-modifying potential. Future research should explore sex differences, long-term outcomes, and adipokines as therapeutic targets in AD. Based on our results we recognize the value of incorporating metabolic data—such as body weight, caloric intake, and insulin levels—as well as brain insulin resistance markers (e.g., phospho-IRS, phospho-AKT, and phospho-GSK3β). As our research progresses toward a more mechanistic understanding, we are actively integrating these additional markers in ongoing studies to further elucidate the interplay between peripheral metabolic states and central adipokine-mediated signaling.

## Revised discussion

Alzheimer’s disease (AD) is a progressive, multifactorial neurodegenerative disorder with no disease-modifying therapies currently available (Cummings et al., 2019; Vaz and Silvestre, 2020). While amyloid and tau pathologies have been central to most therapeutic approaches, recent studies increasingly implicate systemic metabolic dysfunction, including dysregulated lipid and glucose homeostasis, as key drivers of disease onset and progression (Yin, 2023; de la Monte and Tong, 2014; Chew et al., 2020). In this study, we explored whether repurposed metabolic drugs could influence AD pathology using the 5xFAD mouse model, a well-established and aggressive amyloidogenic transgenic line that expresses mutant forms of human APP and PSEN1. This model exhibits early intraneuronal Aβ42 accumulation, amyloid plaque deposition, gliosis, synaptic loss, and cognitive decline by 4–6 months of age, without tau pathology. We tested two FDA-approved agents: Alirocumab, a PCSK9 inhibitor that lowers LDL cholesterol, and Gliclazide, a sulfonylurea that enhances insulin secretion.

The most striking finding was the robust cognitive rescue in 5xFAD mice treated with Alirocumab, as measured by the Y-maze spatial alternation test. This was accompanied by restoration of hippocampal synaptic plasticity, evidenced by normalized input–output responses and significantly improved long-term potentiation (LTP). Additionally, Alirocumab-treated animals exhibited elevated expression of synaptophysin, the neuronal marker β3 tubulin and a reduction in expression of the inflammatory marker TNF-*α*, suggesting preservation of synaptic integrity accompanied by a marked reduction in inflammation. Notably, 5xFAD mice show heightened synaptic responses relative to wild-type (WT) controls, likely reflecting early compensatory hyperactivity in the face of synaptic degeneration. Similar hyperexcitability has been observed in human mild cognitive impairment and in other FAD models, such as those studied by Tang et al. and Huijbers et al., who noted persistent hippocampal overactivity throughout early disease stages (Huijbers et al., 2015; Tang et al., 2016). Alirocumab reduced this aberrant hyperexcitability, implying a shift toward normalized circuit function.

To probe potential mechanisms, we examined brain cholesterol levels across all groups. While HDL and total cholesterol remained unchanged, 5xFAD mice exhibited significantly elevated brain LDL cholesterol, which was fully restored to WT levels following Alirocumab treatment. Although the ability of PCSK9 inhibitors to cross the blood–brain barrier remains under investigation, our data suggest that peripheral lipid modulation can significantly influence central cholesterol profiles, either directly or via systemic feedback loops. This is particularly relevant given accumulating evidence linking high circulating LDL levels to increased AD risk, independent of APOE genotype. By restoring LDL homeostasis, Alirocumab may preserve neuronal membrane composition and function, reduce lipid raft–associated amyloidogenesis, and protect against cholesterol-induced synaptic loss. Furthermore, in terms of glucose regulation, Alirocumab not only normalized insulin levels in 5xFAD mice but also significantly increased IDE expression, pointing to a broader therapeutic impact. This dual action suggests that Alirocumab may influence both metabolic regulation and amyloid degradation pathways.

In contrast, Gliclazide treatment did not yield significant cognitive benefits within the study duration. This is despite a clear reduction in amyloid burden and markers of neuroinflammation, including decreased expression of GFAP, the pan-macrophage marker and TNF-*α* in 5xFAD brains. These findings raise a critical question: if Aβ and gliosis are reduced, why does cognition remain impaired? One possible explanation is that cognitive deficits in 5xFAD mice, particularly by 8–9 months of age, may occur independently of ongoing amyloid and inflammatory pathology, once irreversible synaptic damage has occurred. Alternatively, Gliclazide may exert neuroprotective effects too subtly or too late to yield measurable behavioral improvements in this aggressive model. This aligns with the idea that such metabolic agents may be more effective as preventive rather than restorative therapies—especially given Gliclazide’s modest synaptic and neuronal marker effects (Chen et al., 2011; Rojas et al., 2021).

Importantly, both drugs modulated adipokine expression in the brain, a relatively unexplored mechanism in AD pathophysiology. All four adipokines measured—leptin, adiponectin, omentin, and resistin—were significantly decreased in untreated 5xFAD mice. Alirocumab selectively restored leptin and omentin levels, whereas Gliclazide significantly reduced adiponectin but had no effect on leptin or omentin. Neither drug altered resistin levels. Leptin is of particular interest due to its high receptor expression in the hippocampus and its known role in enhancing synaptic plasticity and modulating tau phosphorylation (Marwarha and Ghribi, 2012; Hamilton and Harvey, 2021). The normalization of leptin following Alirocumab treatment may be one pathway through which synaptic function and cognition are restored. Conversely, the reduction of adiponectin by Gliclazide may reflect systemic metabolic changes without translating to CNS benefits. The complex crosstalk between adipokines, insulin signaling, and CNS integrity warrants further study, particularly in models that include metabolic comorbidities such as obesity or T2D.

To further dissect the relationship between systemic lipid metabolism and brain function, we performed untargeted lipidomics on visceral adipose tissue. Both drugs induced significant remodeling of lipid species, with the most affected subclasses being lysophosphatidylethanolamines (LPE), lysophosphatidylserines (LPS), and lysophosphatidylcholines (LPC) lipids implicated in membrane signaling and inflammation. Pathway analysis identified several lipid metabolism–associated proteins uniquely affected in treated 5xFAD animals, including LPIN1 and DGAT1. We investigated the expression of these proteins in brain tissue to explore potential peripheral-to-central signaling axes.

LPIN1 encodes a phosphatidate phosphatase that regulates the conversion of phosphatidate to diacylglycerol, a precursor for triglyceride synthesis. LPIN1 is essential for maintaining lipid balance and has been linked to hippocampal synapse density and spatial memory performance (Shang et al., 2020). We observed significantly reduced LPIN1 expression in untreated 5xFAD mice, which was restored to WT levels following both Gliclazide and Alirocumab treatment. DGAT1, a triglyceride-synthesizing enzyme that protects against lipotoxic ER stress (Chitraju et al., 2017), was also decreased in 5xFAD mice but restored only by Alirocumab. These findings suggest that key lipid enzymes may link visceral lipid profiles to CNS function, potentially mediating protection against synaptic dysfunction and neurodegeneration.

Taken together, our findings support a growing consensus that AD is at least partially a metabolic disease, with systemic lipid and glucose dysregulation contributing to its progression. While amyloid accumulation remains a defining hallmark, targeting upstream metabolic dysfunction may provide a more effective means of intervention. Alirocumab’s ability to restore cognitive function, synaptic plasticity, adipokine levels, and lipid-related enzyme expression underscores its potential as a disease-modifying therapy. In contrast, Gliclazide’s effects on neuroinflammation and amyloid burden, despite limited cognitive impact, highlight the importance of treatment timing and suggest a role in early-stage or preventive interventions.

Clinically, the fact that both drugs are FDA-approved offers an opportunity for rapid translation. Longitudinal epidemiological studies examining cognitive trajectories in individuals on PCSK9 inhibitors or sulfonylureas could yield valuable insights. Moreover, lipidomic and adipokine markers identified here, particularly LPIN1, DGAT1, and leptin, could serve as peripheral biomarkers for AD risk or therapeutic response. Finally, future work should investigate sex differences, since our study used only male animals, and assess long-term treatment outcomes beyond the 5-month window.

In conclusion, this study provides compelling evidence that systemic metabolic modulation, particularly lipid-lowering via PCSK9 inhibition, can influence AD-related neuropathology and cognitive decline. These findings support a shift beyond amyloid-centric approaches, proposing that metabolic therapies may offer scalable, disease-modifying potential for AD.

Alirocumab and Gliclazide exert distinct but complementary effects on brain function, lipid metabolism, and neuroinflammation. These findings contribute to a growing body of evidence that lipid and glucose metabolism are not peripheral side-issues in AD, but central components of disease pathophysiology. Moving forward, a more integrated approach that includes metabolic profiling, adipokine analysis, and targeted neuroimaging will be critical to fully define the mechanisms by which metabolic health influences neurodegeneration and to unlock new therapeutic strategies for AD.

## Data Availability

The raw data supporting the conclusions of this article will be made available by the authors, without undue reservation.

## Glossary

5xFAD: 5 times transgenic familial Alzheimer’s Disease
AD: Alzheimer’s Disease
ApoE: Apolipoprotein E
APP: Amyloid Precursor Protein
Aβ: beta amyloid
BBB: Blood Brain Barrier
CA1: Cornu Ammonis 1
CA3: Cornu Ammonis 3
CRH: Corticotropin-releasing hormone
DGAT1: Diacylglycerol O-acyltransferase 1
ELISA: Enzyme Linked Immunosorbent Assay
FDA: Food and Drug Administration
fEPSP: field Excitatory Post Synaptic Potentials
GFAP: Glial Fibrillary Acidic Protein
HDL: High-Density Lipoprotein
LC–MS/MS: Liquid Chromatography – Mass Spectrometry
LDL: Low-Density Lipoprotein
LPIN1: Lipin1
LTP: Long-Term Potentiation
PBS: Phosphate-Buffered Saline
PCR: Polymerase Chain Reaction
PCSK9: Proprotein Convertase Subtilisin/Kexin type 9
PSD-95: Post Synaptic Density – 95
SPF: specific pathogen-free
SR: Stratum Radiatum
T2D: Type 2 Diabetes
TRH: Thyrotropin-Releasing Hormone
VAT: Visceral Adipose Tissue
WT: Wild Type
