# Supplementary material for: Systemic lipid and glucose modulation differentially affects cognitive function and neuroinflammation in a mouse model of Alzheimer’s disease
Source: Front Neurosci. 2025 Aug 29;19:1636624. doi: 10.3389/fnins.2025.1636624 (PMC12426080; doi:10.3389/fnins.2025.1636624)
Supplement: Supplementary file 2 [file Supplementary_file_1.docx]

Supplementary Figures and Tables

**
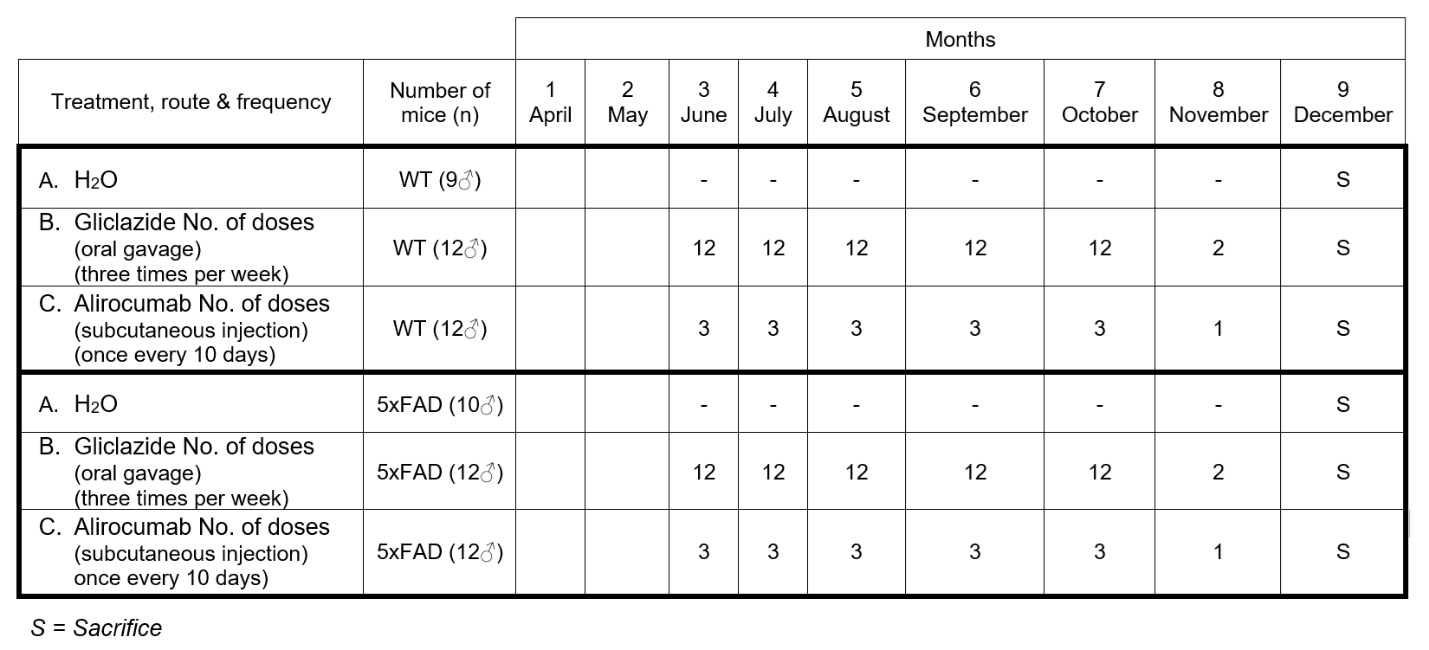
Supplementary Table 1:** Treatment plan. Treatment, delivery route and frequency of drug administration.

**Supplementary Table 2**: Lipid Profile Data. Average values (nmol/mg) and percentage of each lipid group.


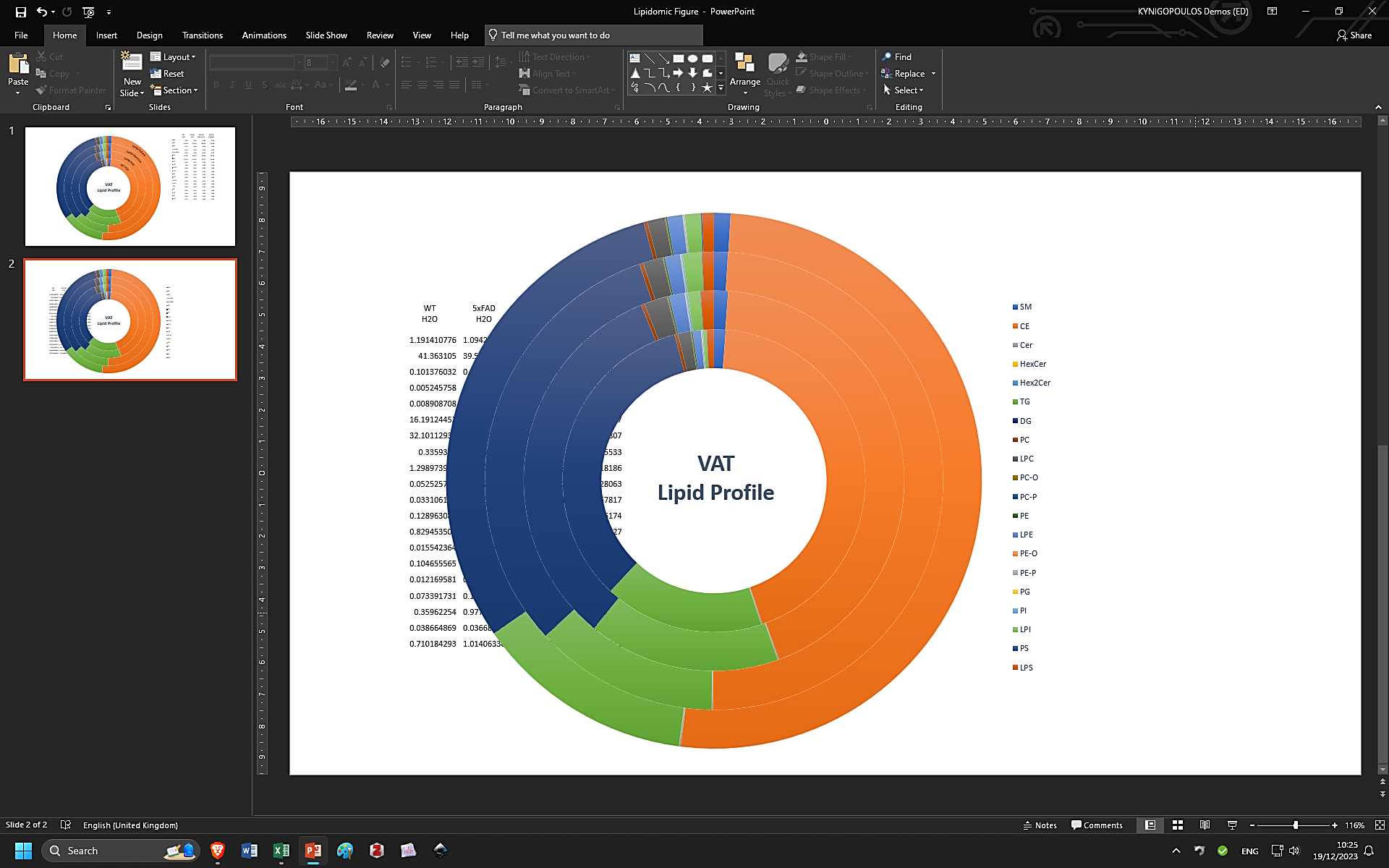

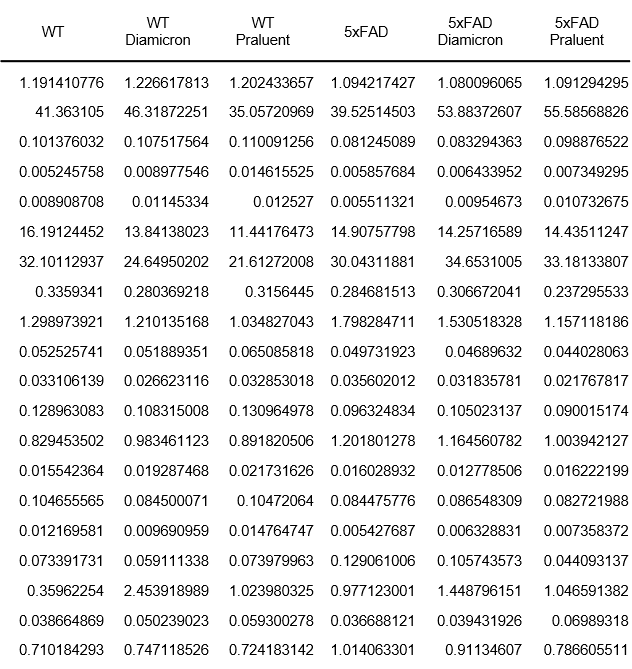


Average Values


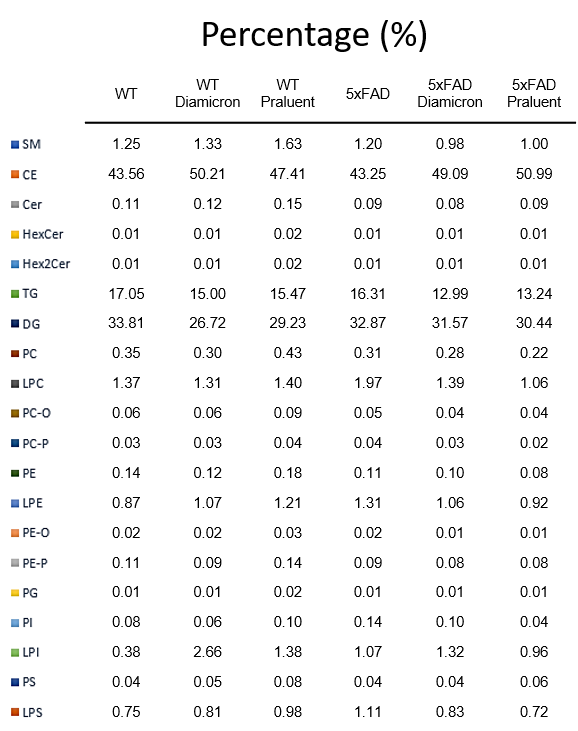


**Supplementary Table 3:** ELISA kits used, with catalog number and type

| **AssayGenie: Technical Manual** | **Catalogue Code** | **Type** |
| --- | --- | --- |
| Mouse Insulin | MOFI00142 | Sandwich ELISA |
| Mouse Insulin Degrading Enzyme (IDE) | MOEB1762 | Sandwich ELISA |
| Mouse HDL (High Density Lipoprotein) | MOFI00888 | Sandwich ELISA |
| Mouse LDL (Low Density Lipoprotein) | MOFI00954 | Sandwich ELISA |
| Mouse TNF alpha | MOFI00104 | Sandwich ELISA |
| Mouse Leptin | MOFI00070 | Sandwich ELISA |
| Mouse ADP/Acrp30 (Adiponectin) | MOFI00001 | Sandwich ELISA |
| Mouse ITLN1 (Intelectin 1/Omentin) | MOFI00950 | Sandwich ELISA |
| Mouse RETN (Resistin) | MOFI00093 | Sandwich ELISA |
| Mouse Interleukin 6 (IL6) | MODL00714 | Sandwich ELISA |
| Mouse Corticotropin Releasing Hormone (CRH) | MODL00309 | Competitive ELISA |
| Mouse Thyrotropin Releasing Hormone (TRH) | MODL01334 | Sandwich ELISA |
| Mouse Glucose Transporter 1 (GLUT1) | MOFI00856 | Sandwich ELISA |
| Mouse Glucose Transporter 4 (GLUT4) | MOFI00857 | Competitive ELISA |
| Mouse Tubulin beta-3 chain (Tubb3) | MOEB1581 | Sandwich ELISA |
| Mouse CD68 / Macrosialin | MOFI00564 | Sandwich ELISA |
| Mouse Synaptophysin | MOFI01130 | Sandwich ELISA |
| Mouse Glial Fibrillary Acidic Protein (GFAP) | MOEB0065 | Sandwich ELISA |
| Mouse C-reactive protein (Crp) | MOEB0618 | Sandwich ELISA |
| Mouse Diacylglycerol O-acyltransferase 1 (DGAT1) | MOFI01208 | Sandwich ELISA |
| Mouse Lipin1 (LPIN1) | MODL00820 | Sandwich ELISA |
| **Invitrogen: Technical Manual** | **Catalogue Code** | **Type** |
| Mouse Aβ40 | KMB3481 | Sandwich ELISA |
| Mouse Aβ42 | KMB3441 | Sandwich ELISA |

**Supplementary Figure 1:**


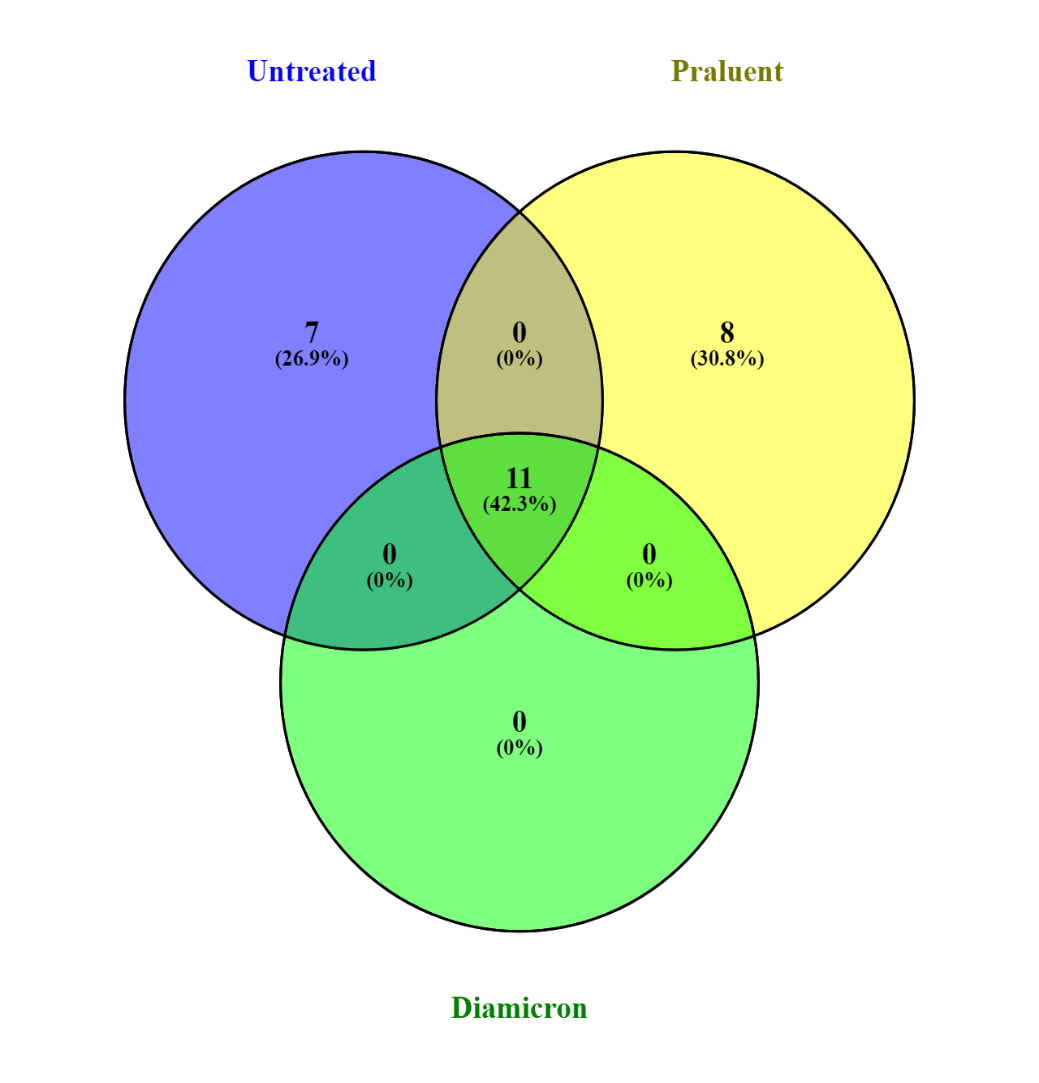


*

Lpin1, Lpin2, Lpin3, Dgat1, Plcz1, Plch2, Plcd3, Mfsd2a

Lpcat3, Atp8a1, Atp8b3, Plscr1, Ptpmt1, Lpcat1, Abhd16a

Abcb1a, Abcb4, Ano3, Ano4, Ano6, Ano7, Ano9, Hrasls5, Pltp, Mtp, Mtmr7

*

**Figure S1:** Venn Diagram showing the significant proteins between the groups. Eleven proteins are found to be shared between all three groups. The untreated group revealed seven unique proteins, whereas the Praluent group revealed eight. No additional unique or shared proteins are to be observed. Figure generated from https://bioinfogp.cnb.csic.es/tools/venny/index.html

**Supplementary Figure 2:**


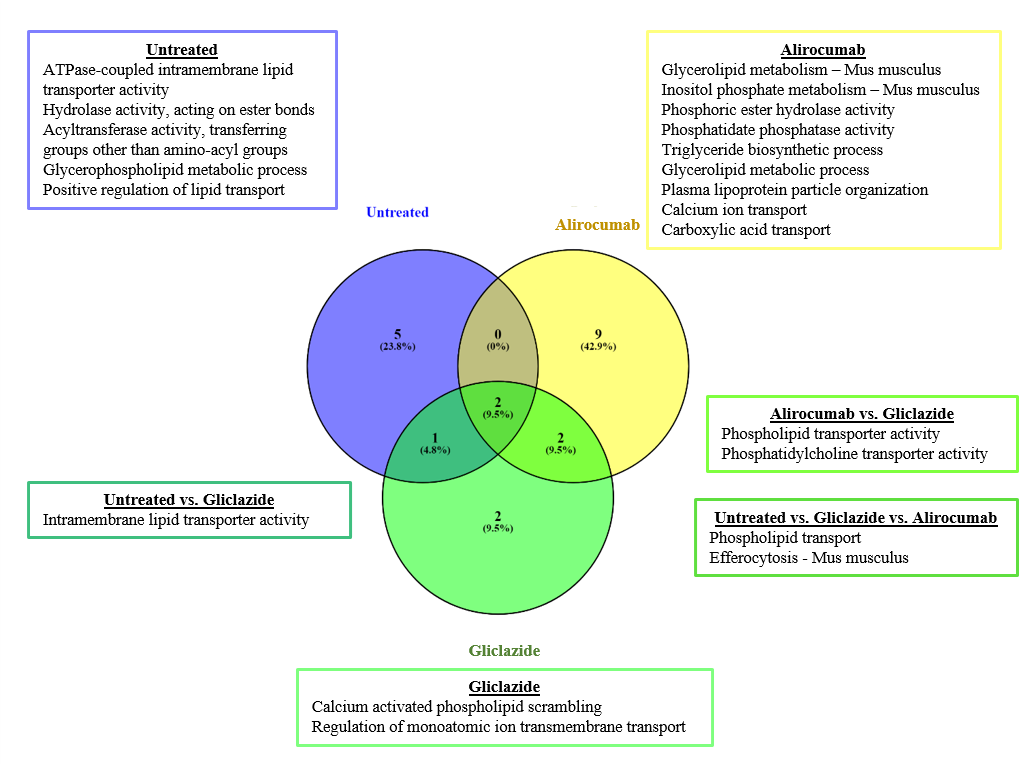


**Figure S2:** Venn Diagram showing the significant pathways generated by MetaScape. The untreated group revealed five unique pathways, the Alirocumab had nine unique pathways and the Gliclazide had two unique pathways. Two pathways are found to be shared between all three groups, one between untreated and Gliclazide and two between Alirocumab and Gliclazide. Figure generated from https://bioinfogp.cnb.csic.es/tools/venny/index.html


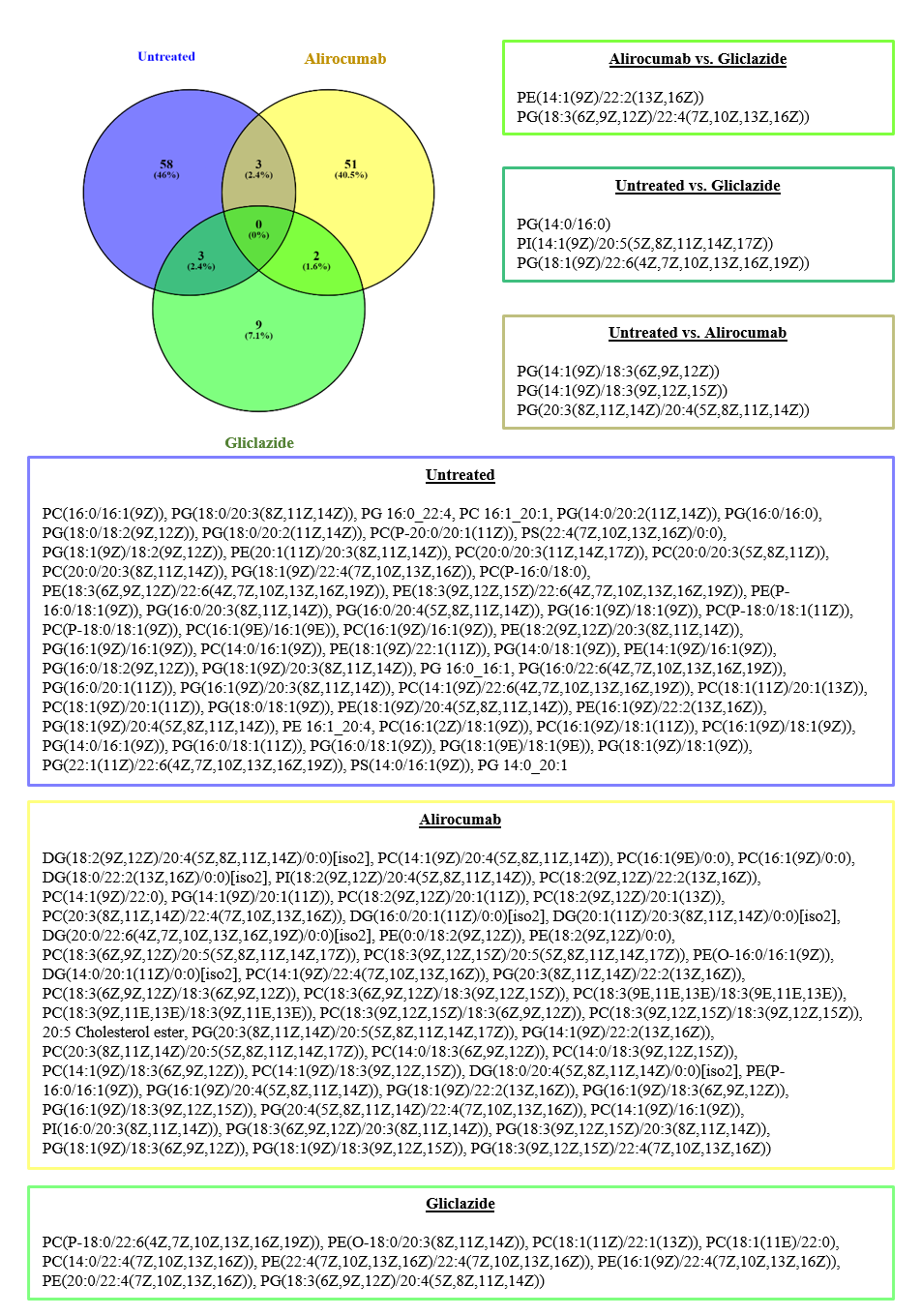
**Supplementary Figure 3:**

**Figure S3:** Venn Diagram showing the significant lipids generated by LipidMaps. The untreated group revealed fifty-eight unique lipids, the Alirocumab had fifty-one unique lipids and the Gliclazide had nine unique lipids. Three lipids are found to be shared between untreated and Alirocumab, three between untreated and Gliclazide and two between Alirocumab and Gliclazide. No lipids were found to be shared between all three groups. Figure generated from https://bioinfogp.cnb.csic.es/tools/venny/index.html

**
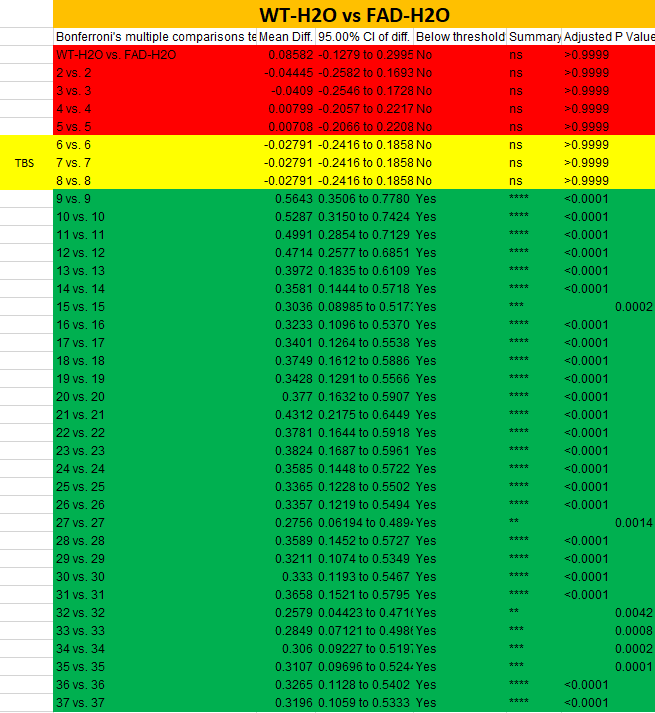
Supplementary Table 4**: Statistical analysis of LTP electrophysiology data. One-way ANOVA and Bonferroni post hoc test. The non-significant values are marked with red and the significant with green. With yellow is the Theta Burst Stimulation

**
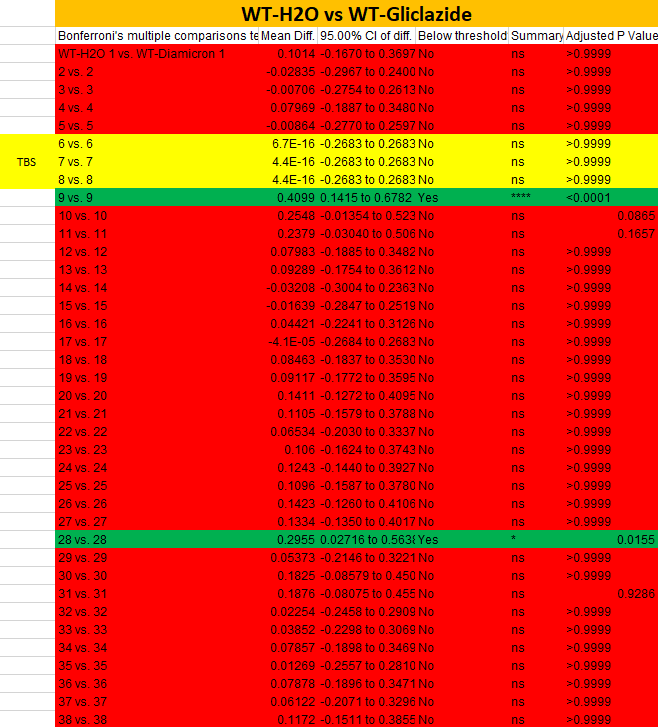
Supplementary Table 4:** continued

**
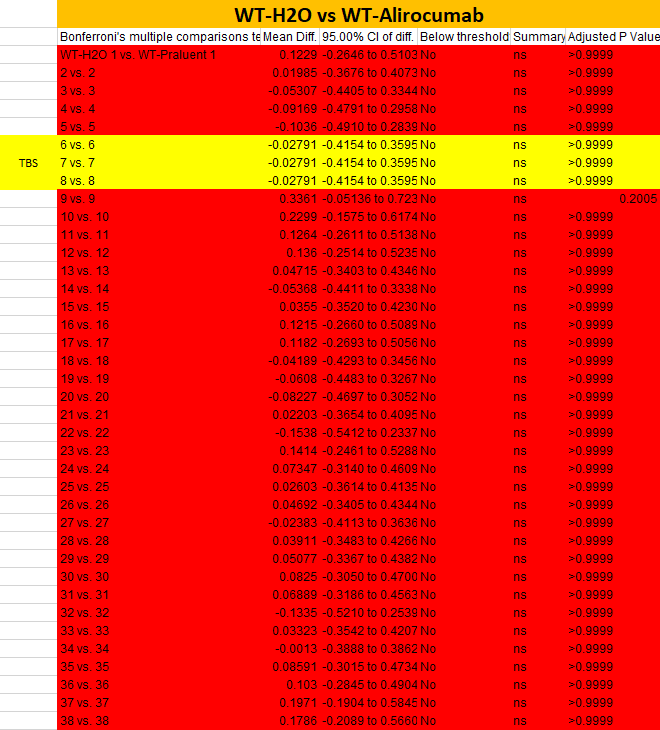
Supplementary Table 4:** continued

**
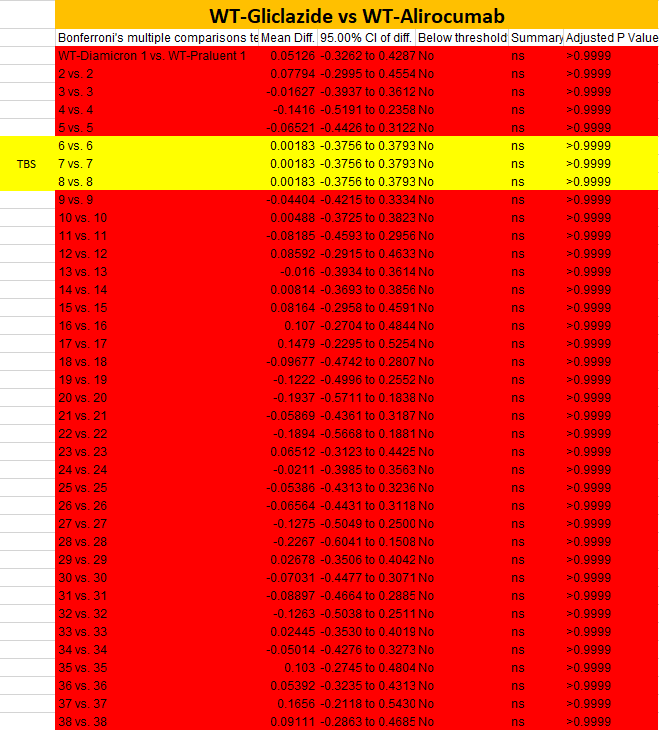
Supplementary Table 4:** continued

**
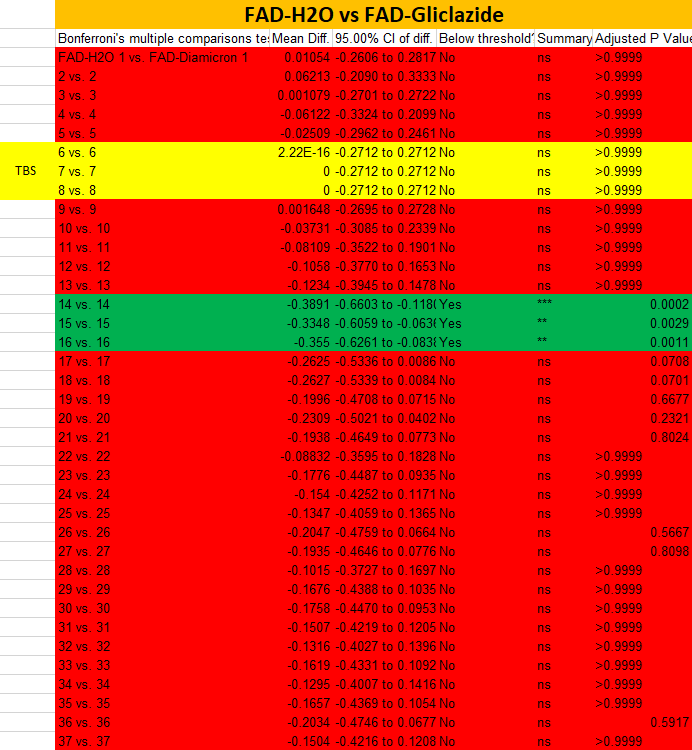
Supplementary Table 4:** continued

**
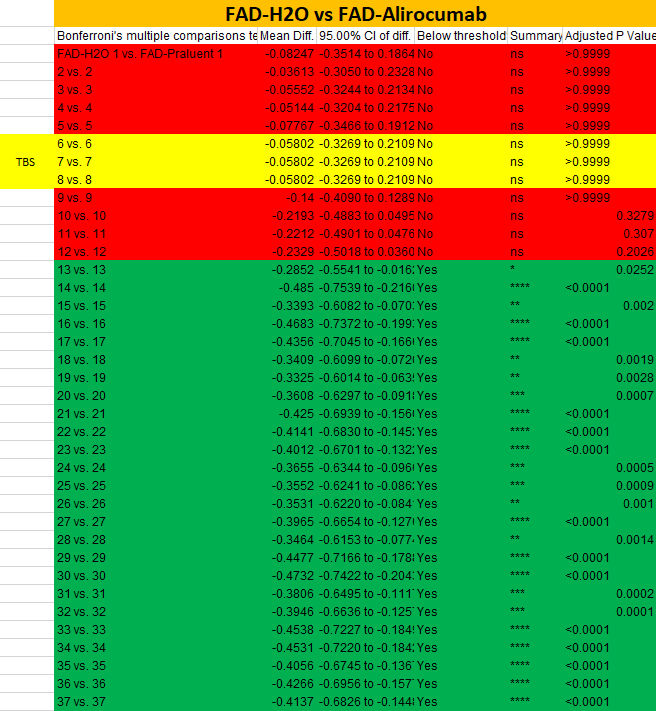
Supplementary Table 4:** continued

**
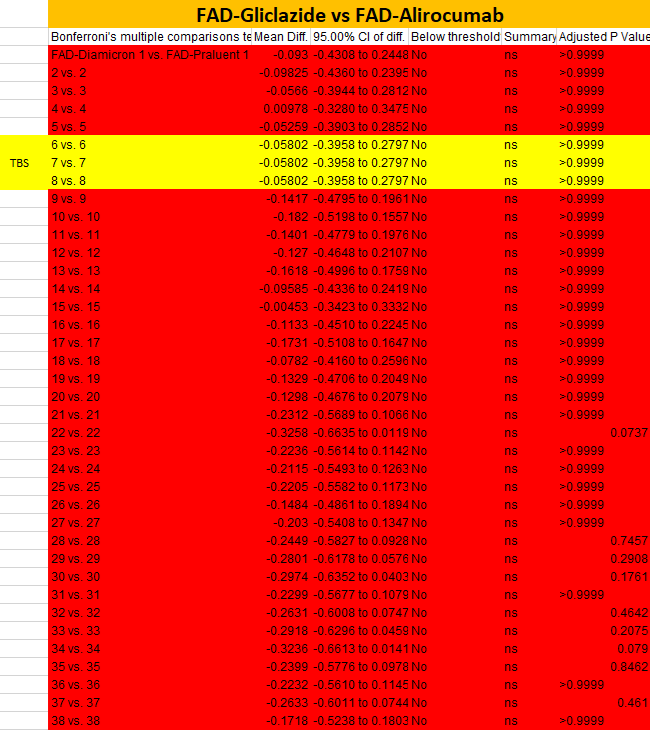
Supplementary Table 4:** continued

**Supplementary Table 5:** Statistical analysis of I/O electrophysiology data. Two-way ANOVA and Bonferroni post hoc test. The non-significant values are marked with red and the significant with green.


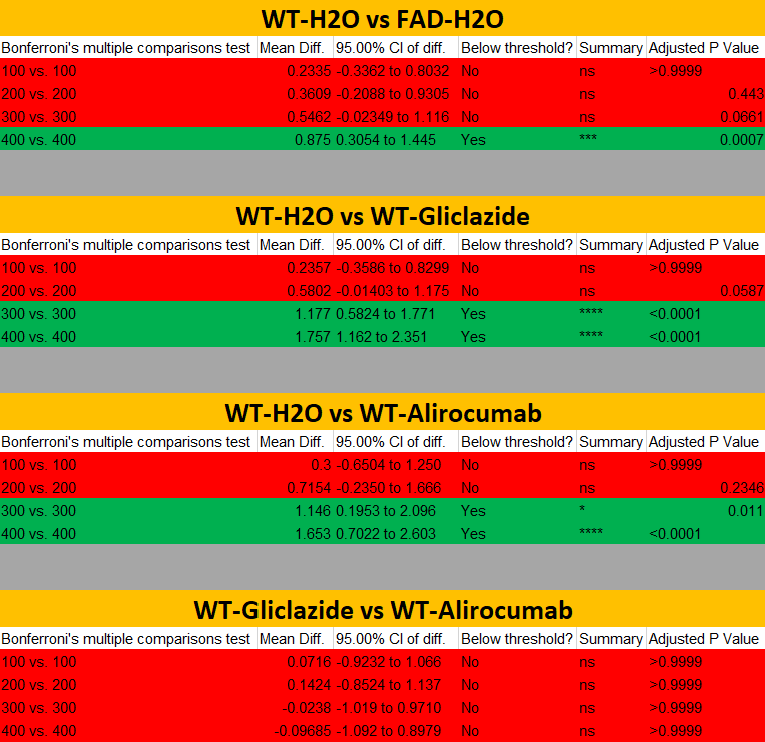

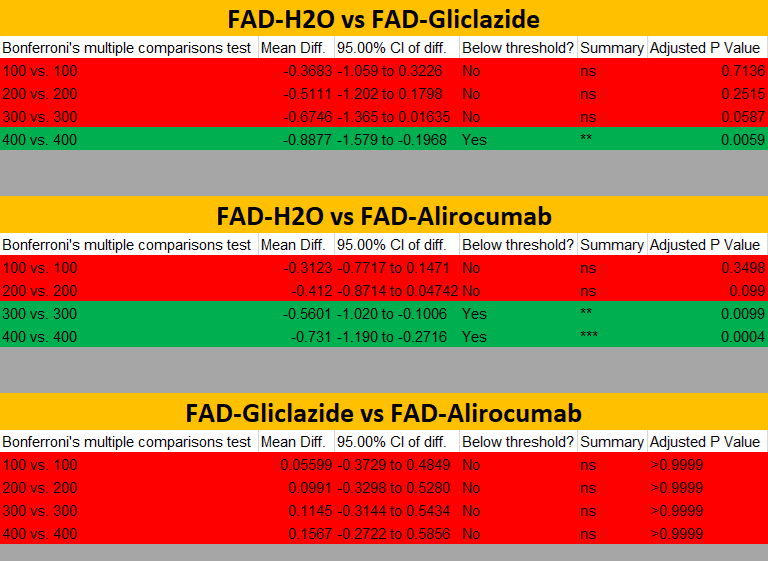


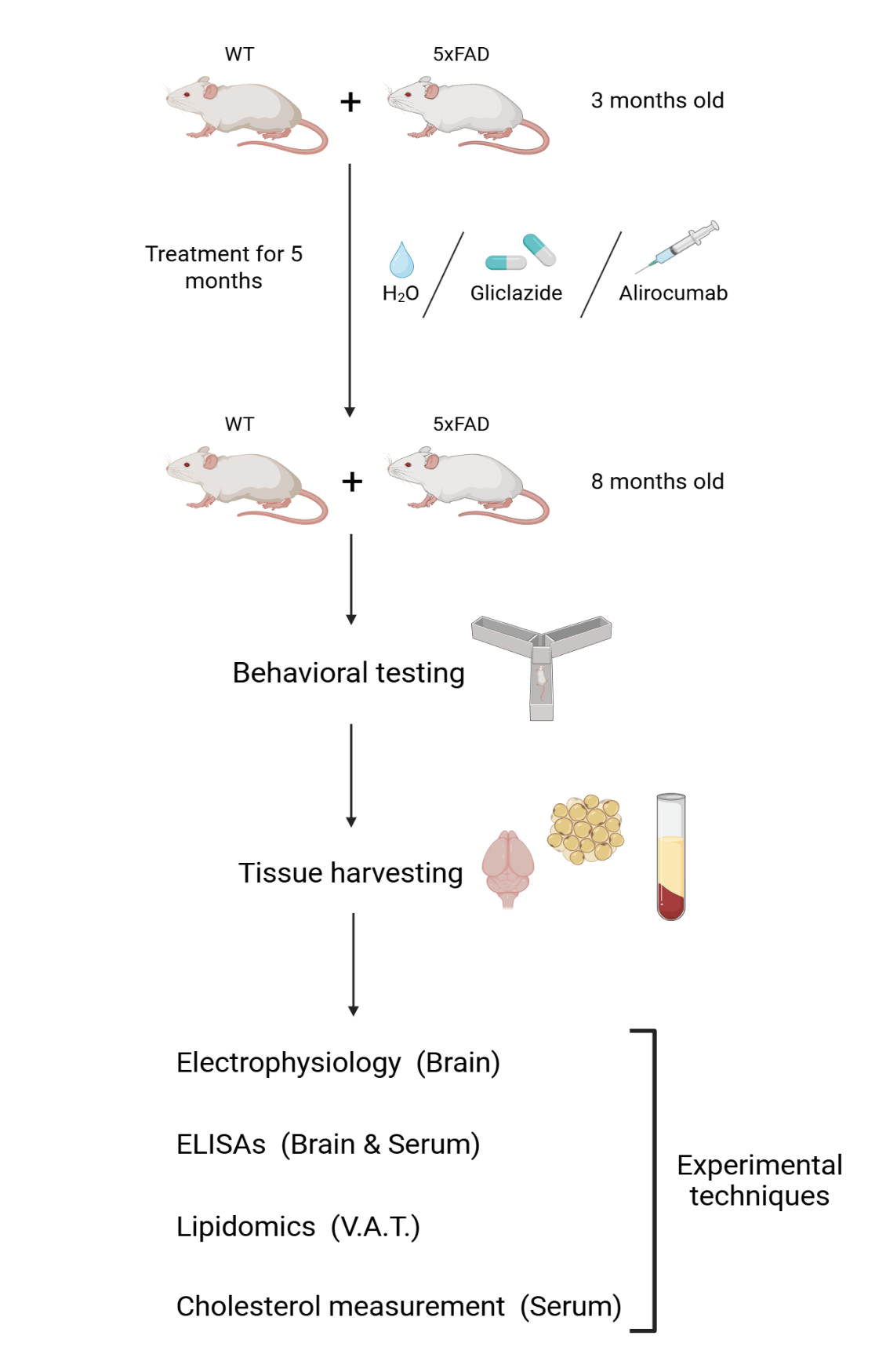
**Supplementary Figure 4:**

**Figure S4: Schematic representation of the experimental workflow. The figure shows the order the experiments took place. First, the mice were genotyped and separated into 5xFAD and WT groups. Both groups were sub-divided again depending on their treatment protocol (control (water), Gliclazide, Alirocumab). Following five months of treatment, the behavioral test was carried out and tissues were then harvested. Electrophysiology, immunoassays, lipidomics and cholesterol measurements were finally carried out. All analyes were performed on the same tissue. Created in Biorender (https://BioRender.com)**
